# Nanoparticle-Based Antigen Delivery: Advancing Immunization Strategies against Infectious Pathogens

**DOI:** 10.34133/bmr.0385

**Published:** 2026-07-03

**Authors:** Surajit Patra, Nida Irfan Pathan, Yogesh A. Karpe, Virendra Gajbhiye

**Affiliations:** ^1^ Nanobioscience Group, Agharkar Research Institute, Pune 411004, India.; ^2^ Savitribai Phule Pune University, Pune 411007, India.

## Abstract

Stimulation of adaptive immunity is a goal to defend against infectious agents. In recent years, vaccine development and improvements in their efficacy have been thoroughly investigated, and highly effective vaccines have been released to the market. The administration of vaccinations, safety, and the development of immunogenicity pose considerable challenges in this area. Investigations have been conducted on nanoparticle (NP)-based vaccines to address the issue. NPs are used in vaccine development to enhance antigen delivery, provide protection, and serve as adjuvants. The application of nanotechnology has substantially improved the delivery and effectiveness of vaccines by manipulating NP properties. Studies on NP-based immunizations have focused on viral pathogens and bacterial agents. Immunizations utilizing NPs enhance immune responses and stabilize viral antigens, facilitating the development of vaccines for hepatitis B, influenza, and HIV. Nanovaccines specifically target antigens associated with viruses, bacteria, and certain cancers. NP-based vaccines effectively stimulate and mature dendritic cells (DCs) in vitro and in vivo. Following vaccination, the NP-based vaccine resulted in increased cytokine levels and activated Th1 and Th2 cells in vivo. As a result, NP-based vaccines stimulate T-cell immune responses and adaptive immunity driven by immunoglobulin G and immunoglobulin M. This review has discussed the various NPs and antigens used in nanovaccine preparation; the role NPs play in activating adaptive immunity; and, specifically, the maturation of DCs; the activation of Th1 and Th2 cells against viruses, bacteria, and other microbial pathogens; and the safety of NPs.

## Introduction

The history of vaccination and immunization begins with the experiment by Dr. Edward Jenner in 1796, where he injected pus from a cowpox lesion obtained from an infected milkmaid’s hand into an 8-year-old boy [1]. The experiment’s outcome influenced the scientific world to develop vaccines against life-threatening diseases. The development of the vaccine, the most marked successful public health endeavor to date, has helped save many lives. The “isolate, inactivate, and inject” paradigm of Louis Pasteur served as the foundation for the vaccine’s development [2]. The Food and Drug Administration (FDA) has approved 88 vaccines against 32 infectious agents [3]. However, still no effective vaccine is available against many diseases, such as HIV [4–7], dengue [8,9], malaria [10], and tuberculosis (TB) [11,12]. Among the different types of vaccines, live-attenuated vaccines (LAVs) have a chance of reversion (safety issue) after vaccination [13]. LAVs can prevent viral diseases such as smallpox, polio, measles, mumps, and yellow fever. Viruses are produced by propagating a virus in cultured cells. Numerous LAVs exhibit a reversion to virulence. This may occur due to back-mutation of attenuating mutations, correction of mutations in other genomic regions, recombination or reassortment, or alterations in quasispecies diversity. The mixing of numerous LAVs may influence their virulence or immunogenicity by either enhancing or competing with the individual vaccine viruses [14,15]. LAVs provide protection against 3 bacterial and 12 viral diseases. Self-resolving infections may arise from partial immunodeficiency. Genetic immunodeficiencies result in severe LAV infections. In 1951, 1963, 1966, and 2009, BCG, OPV, vMeV, and ORV have all been associated with inborn errors of immunity (IEIs). Notwithstanding genetic variation, these 4 LAVs exhibited immunologically similar IEIs. Congenital deficiencies in IFN-α/β and IFN-λ immunity result in vMeV pathology, adaptive immunity leads to ORV pathology, IFN-γ immunity induces BCG pathology, and B-cell immunity precipitates OPV pathology. Research must concentrate on severe reactions to the remaining 11 LAVs, presently classified as “idiopathic” owing to the absence of genetic or acquired immunodeficiencies. The investigation of IEIs that induce life-threatening LAV infections is immunologically pertinent for understanding host defense against both parental and attenuated viruses, and it holds clinical significance for patients and their families [16]. At the same time, other vaccines, such as recombinant (DNA, RNA) vaccines, inactivated vaccines, and toxoid vaccines, help to induce immunity against the antigen. However, as compared with LAVs, the immune cell may not stimulate adequately, so booster doses or adjuvants may be needed after a few vaccination periods [17,18]. However, numerous challenges persist in the vaccine development process, encouraging scientists to continue to create better and innovative vaccine technologies. Substantial advancements have been made in the development of vaccines [19,20]. Today, we are in the era of contemporary vaccinations, which means that the modern concept is used for the production of vaccines, vaccine development is moving faster than ever, and the utilization of adjuvants and innovative delivery systems increases immunogenicity [21].

Nanotechnology transforms medication and vaccine delivery methods in medical sciences by precisely regulating nanoparticle (NP) size, shape, surface qualities, and composition. This enables the development of next-generation pharmaceuticals and vaccines that can specifically target and interact with specific cells in the body [22,23]. Because of their small size, NPs can quickly enter living cells by pinocytosis or cellular endocytosis [24]. The global nanotechnology market in 2020 is 42.2 billion US dollars, and it will reach a revised size of 70.7 billion US dollars in 2026 [25]. To advance past mere descriptive categorization of NP platforms, nanovaccines must be engineered with design parameters that profoundly influence immune responses. These parameters include size, shape, surface charge, surface chemistry, targeting ligands, endosomal escape capability, release kinetics, adjuvant codelivery, and route of administration. NPs ranging from 10 to 100 nm migrate to draining lymph nodes and exhibit superior antigen presentation compared to larger particles [26,27]. The surface charge of a cell influences the absorption and mobility of materials. Cationic systems enhance cell absorption; however, they also lead to increased inflammation and cell death [26]. Studies indicate that the composition of ionizable lipids in messenger RNA (mRNA) vaccination systems influences endosomal escape and CD8^+^ T-cell activation [28]. The extended-release kinetics of biodegradable polymeric nanoparticles (PNPs) enhance germinal center formation and sustain long-lived plasma cell responses compared with traditional bolus antigen delivery [29]. Surfaces modified with targeting ligands have the potential to enhance the delivery of antigen-presenting cell (APC) subsets, thereby influencing immunological polarization [30]. Synthetic NP systems incorporating Toll-like receptor (TLR) agonists facilitate the simultaneous delivery of antigens and immunostimulatory molecules, thereby activating both innate and adaptive immune responses [31]. The injection method influences NP design, lymph node targeting, and the specificity of tissue immunity [26]. In light of these experimental findings, it is essential to develop nanovaccine platforms by actively manipulating their physicochemical characteristics to align with the immunological requirements of pathogens, rather than relying solely on material classification.

Even with the increasing number of studies and assessments regarding nanovaccines, crucial gaps remain in understanding the mechanisms that govern the interaction between immune cell activation, translational effects, and the design parameters of NPs. Recent studies have highlighted advancements in antigen delivery, immunomodulatory platforms, and multifunctional carrier systems, including liposomes, PNPs, nanogels, and lipid-based systems, for applications in infectious diseases, cancer immunotherapy, and mucosal vaccination procedures [26–29,32]. Additional investigation has focused on clinical translation and design challenges, highlighting the need for formulation adaptability while acknowledging barriers to broader implementation [30,31].

Despite this comprehensive investigation, most research primarily emphasizes application domains or platform categorization. Their ambiguity regarding the influence of specific physicochemical traits—such as size, surface charge, architecture, spatial arrangement of antigen–adjuvant, and endosomal escape capacity—on dendritic cell (DC) uptake, macrophage activation, antigen processing pathways, lymphatic trafficking, and the activation of CD8^+^ T cells and B cells is evident. The connection between strategic vaccine development and the mechanistic interactions between NPs and immune cells remains insufficiently understood.

Our study offers a clear, mechanism-oriented framework that links NP design principles to immune cell-specific activation pathways and disease-targeted strategies, while also taking into account translational and regulatory considerations [30,31]. This study links nano-immune interactions to practical challenges in vaccine development, moving past simple classification of nanovaccines to focus on informed immunological engineering.

## Why NPs for Vaccine Delivery?

In traditional vaccines, the preparation of live attenuated microbes, inactivated microbes, killed microbes, or parts of microbes is used. Some of these vaccines give a good immune response against microbial infection, but some fail to do so. Sometimes, these traditional live vaccines are capable of causing disease in immunocompromised individuals. Another problem in vaccine development is age-related immunosenescence, which means fewer APCs like DCs in the blood and tissues, and also overall decreased phagocytosis and TLR signaling, leading to chronic inflammation in elderly individuals. Immunosenescence is the major problem in traditional vaccine preparations. The effectiveness of vaccines substantially declines in older adults due to immunosenescence, which includes thymic involution, reduced naive T- and B-cell repertoires, compromised DC antigen presentation, and persistent low-grade inflammation (inflammaging). The outcomes of these age-related impairments include reduced T-cell priming, diminished germinal center responses, and decreased antibody affinity maturation following routine immunization [33,34].

Nanotechnology may offer a promising approach for overcoming challenges in vaccine development. It could allow for the incorporation of a greater number of antigens, facilitate more effective booster doses, support the potent action of adjuvants, and enable the precise, targeted delivery of antigens specifically to APCs.

Additionally, many infectious diseases have no licensed vaccine; for these diseases, recombinant DNA technology (RDT) is used for vaccine development. The isolated RDT product, such as proteins, polysaccharides, or recombinant DNA, is used for protection against the antigen. These RDT products are more defined, safer, and less reactogenic than the traditional vaccine. However, the RDT product used in vaccine development required adjuvants to boost efficacy. Thus, aluminum salt-based adjuvants are generally used. Nevertheless, they may fail to generate cell-mediated solid immunity [35–37].

### Role of NP-based vaccine in immune cell activation

The use of NP-mediated vaccine delivery can overcome this problem. NPs, 1 to 1,000 nm in size, were efficiently internalized by APCs, such as DCs and macrophages. NPs inhibit enzymes from degrading antigens, thus ensuring that the immune system were exposed to the complete antigen. Antigens may be delivered into the cytoplasm of APCs via NPs by escaping endosomes. The main mechanism for combating infections involves the activation of CD8^+^ cytotoxic T lymphocytes (CTLs) through major histocompatibility complex (MHC )class I presentation. NPs can be designed to incorporate both immune-enhancing compounds, such as TLR agonists, and antigens. This results in the activation and maturation of DCs, subsequently inducing a Th1-biased response that emphasizes cell-mediated immunity rather than humoral (antibody) responses. Because of their small size, NPs were also transported to lymph nodes, where they activate T cells. This promotes the interaction between naive T cells and the pathogen, resulting in the generation of robust memory T cells that provide long-term protection. Antigens may be released by NPs either gradually or in a single event, akin to natural infections. Prolonged exposure to antigens strengthens T-cell responses. CD4^+^ Th1 cells and CD8^+^ T cells were activated. Memory T cells are designed to confer long-term protection to the body from pathogens. This approach is beneficial for treating intracellular pathogens, such as viruses, bacteria, and protozoa [32,38,39]. Lipid-based nanovaccines, especially lipid nanoparticles (LNPs) designed for nucleic acid delivery, can enhance lymph node trafficking, boost cytosolic antigen expression and activate innate immune pathways such as TLR and retinoic acid-inducible gene I signaling. These advancements may help mitigate existing limitations and enhance humoral responses and T follicular helper cell induction, even in older populations [38–40]. PNPs, such as poly(d,l-lactic-co-glycolic acid) (PLGA)-based systems, enable the codelivery of immunostimulatory adjuvants that enhance costimulatory signaling. They address age-related deficiencies in DC maturation and antigen persistence by ensuring sustained antigen release and promoting uptake by professional APCs [41,42]. Given that older individuals possess reduced naive T-cell pools and compromised cross-presentation, which hinder conventional vaccine efficacy, nucleic acid-based nanovaccines, including mRNA and DNA platforms, facilitate in situ antigen production and robust activation of CD4^+^ and CD8^+^ T cells [43,44].

### Advantages of using NPs in vaccine delivery

Modern vaccination methods use NP-based vaccine delivery technologies to target immunogenicity and overcome biological limitations of soluble antigens. NPs can protect structurally fragile antigens, such as recombinant proteins, peptides, and mRNA, from rapid enzymatic degradation. Encapsulation in lipid or polymeric nanocarriers protects conformational epitopes, protects antigens from nucleases and proteases, and increases the accessibility of immunostimulatory sites. LNP mRNA vaccines rely on this idea. Clinical translation and foundational investigations have shown that mRNA-LNP platforms were more effective for mRNA delivery, as evidenced in the COVID-19 vaccine development [28,40,41].

APCs efficiently ingest pathogen-mimetic NPs (20 to 200 nm) via phagocytosis and endocytosis, thereby boosting immune responses. Experimental results show that particulate antigens are better absorbed than soluble antigens, increasing T-cell priming cytokines and costimulatory molecules [42,43]. Dimensions affect lymphatic trafficking. NPs (10 to 100 nm) travel to lymph nodes and activate naive T and B cells. In vivo tracking tests show that NPs target lymph nodes better than soluble antigens. This increases adaptive immune responses [27,43].

NPs can deliver immunostimulants or act as adjuvants. Surface charge and chemical composition affect DC growth, innate immune recognition, and inflammasome activation. Polymeric and inorganic NPs directly activate innate pathways that produce cytokines [31,44]. Instead of administering adjuvants and antigens separately, NPs enable in-cell delivery. Trials demonstrate improved CD8^+^ T-cell responses and cross-presentation [30,45]. Endosomal escape is necessary for nucleic acid vaccinations and cytotoxic T-cell activation. Ionizable lipids and pH-responsive polymers breach endosomal membranes to transfer cytosolic contents to improve mRNA translation and MHC-I antigen presentation. LNP systems show that lipid content and *p*K_a_ changes affect endosomal escape efficiency and immunization efficacy [41,46,47]. NPs can alter antigen delivery, boosting immunological response. Preclinical studies show that controlled-release PLGA NPs increase germinal center growth, antigen exposure, and antibody maturation [5]. Finally, tailored administration and immunogenicity allow for fewer doses, improving therapeutic safety. Smaller antigen concentrations can still induce protective immunity while minimizing systemic exposure [40,41]. NPs can change antigen mobility and stability, modulate innate immune perception, and program the adaptive immune system, proving they are more than just transporters.

The NPs possess a substantially higher surface-to-volume ratio for maximum antigen delivery with minimum vaccine dosage. Also, the NPs can protect the antigen from degradation in extracellular environmental conditions. This will allow extended circulation within the body, leading to maximum exposure to the immune system. Other advantages of nanotechnology include site-directed antigen delivery to immune cells, which may increase immune response. Also, vaccines can be delivered through various routes of administration. Moreover, combining several antigens on the same NPs protects against multiple diseases.

## Types of NPs Used in Vaccine Delivery

Vaccines represent one of the most marked advancements in public health. The vaccine technology (conventionally focused on either live attenuated or inactivated vaccines) has prevented morbidity and mortality every year. However, the application of nanotechnology to vaccine development revolutionized this field. NPs have emerged in both academia and the pharmaceutical industry as promising carriers for the development of future vaccines, called nanovaccines [35]. Herein, the NPs can be tethered to or synthesized with components that can stimulate the host’s immune system. This is possible because NPs possess intrinsic adjuvant properties and can boost the immune response, promoting rapid, enduring humoral and cellular immunity. Therefore, given their potential benefits, such as site-specific antigen delivery, enhanced antigen bioavailability, and reduced side effects, several NP-based vaccines are being developed, including inorganic and PNPs, liposomes, micelles, immunostimulatory complexes, virus-like particles (VLPs), and extracellular vesicles (EVs) [36].

### Inorganic NPs

**Fig. 1.**
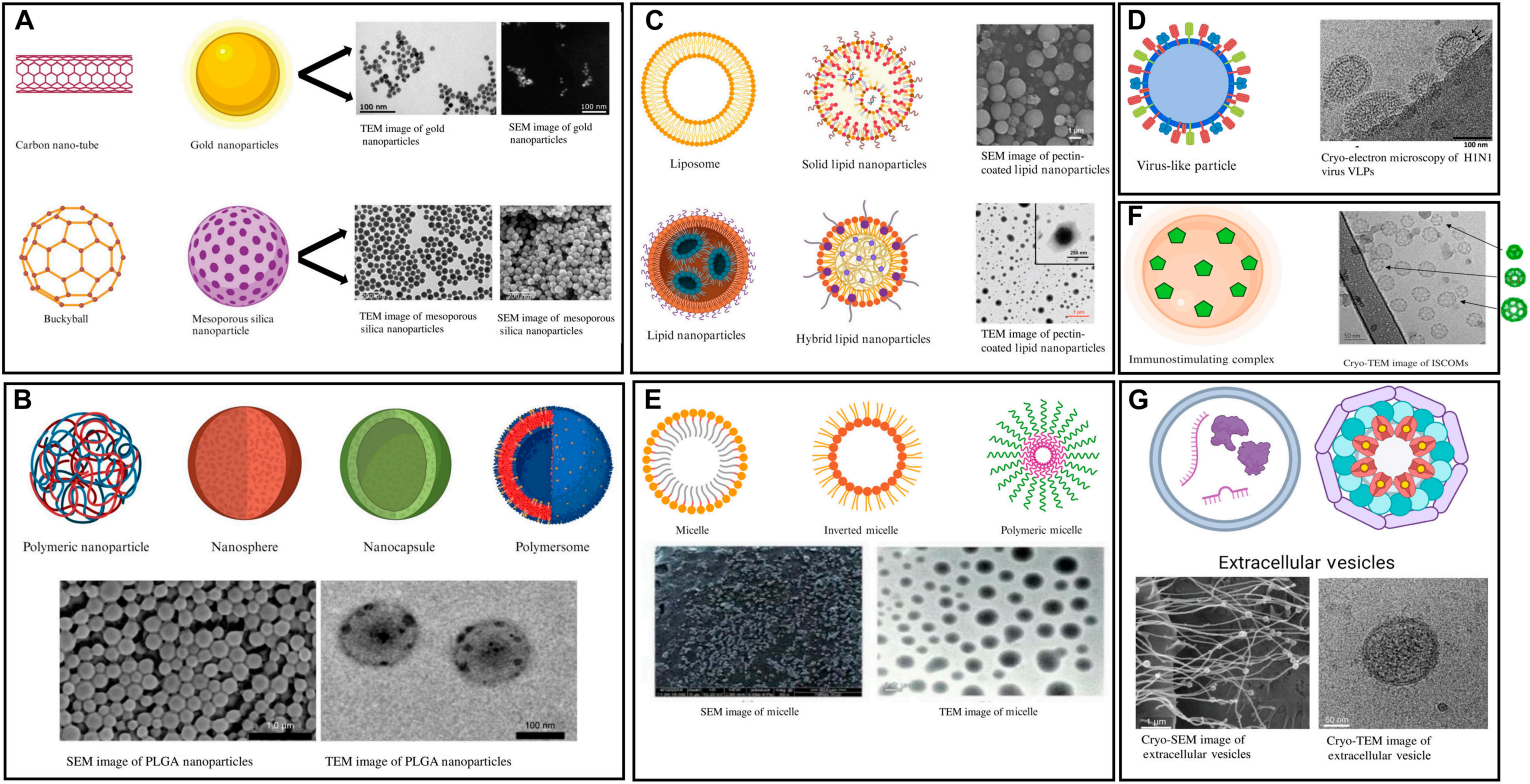
Different types of NPs with their SEM and TEM structures. (A) Structure of different types of inorganic NPs. Gold (Au1) NPs images TEM and SEM image. Silica (Si2) NPs TEM and SEM image. Reprinted with permission from Eaton et al. [48]. Copyright (2017) Elsevier. (B) Different types of polymeric NPs. SEM and TEM image of PLGA NPs. This figure is reproduced under the terms of the Creative Commons CC BY license. Copyright (2017) Dove Medical Press Limited [59]. (C) Structure of different types of liposomes. SEM and TEM image of LNPs. This figure is reproduced under the terms of the Creative Commons CC BY license. Copyright (2017) Nature [90]. (D) Structure of virus-like particle. Image by cryo-electron microscopy of a field of VLPs. Arrows denote some protruding spikes on the surface on a particle. The particles have oval-shaped outlined perimeters at the base of the protruding spikes. A portion of this outline in one particle is denoted by a hashed arc near the base of the indicated spikes (right particle). Scale bar, 100 nm. This figure is reproduced under the terms of the Creative Commons CC BY license. Copyright (2018) Nature [104]. (E) Structure of micelle. Scanning electron microscopy image of prepared micelles, and transmission electron microscopy images of micelle. Reprinted with permission from Bhardwaj et al. [111]. Copyright (2014) Royal Society of Chemistry. (F) ISCOMs and cryo-TEM image of ISCOMs in suspension visualized by cryo-TEM. The scale bar is 50 nm. For comparison, the ISCOM matrix structures derived from the SAXS data are also shown. Reprinted with permission from Pedersen et al. [115]. Copyright (2012) Elsevier. (G) Extracellular vesicle structure. Conventional SEM image of tubular extensions from HAS3 cells containing spherical structures along their lengths. Cryo-TEM of frozen-hydrated vesicles post-isolation from HAS3 cells shows heterogeneity in vesicle shape and size. Reprinted with permission from Noble et al. [127]. Copyright (2020) Elsevier.

Vaccine administration often involves inorganic NPs ranging in size from 2 to 150 nm and are nonbiodegradable due to their rigid structure and easily controlled synthesis (Fig. 1A). Aluminum salt base, gold, silica, and calcium phosphate are examples of inorganic NPs [48–51]. For instance, the influenza A virus surface membrane protein-loaded gold nanoparticle (AuNP) induces a cell-mediated high level of antibody generation and protects from the influenza A virus in the mouse model [52]. Surface-modified mesoporous silica NPs (MSNPs) are an effective medication delivery method. MSNPs also deliver siRNA and DNA safely and precisely to create vaccines [53–55]. Inorganic NPs are used as adjuvant and delivery vehicles for antigens in vaccine development to generate cell-mediated and humoral immunity. Aluminum NPs are generally used as an adjuvant for antigen-specific strong immune response generation in the vaccine. In a few studies, NP-based adjuvants improved the immune response and transportation of protein and peptide antigens for fighting viral infections. These adjuvants comprised sphere versions of carbon NPs, hollow MSNPs, and nanotubes. Moreover, numerous silanol groups found in silica-based NPs can be used to bring particular functional groups to their surface, allowing vaccine molecules to enter target cells [56–58].

### Polymeric NPs

Nanovaccine engineering is flexible and uses PNPs. These nanocarriers’ efficiency depends more on polymer chemistry than particle size, unlike many others. Polymers differ in their breakdown, immune system activation, and cell-drug delivery. The properties of PNPs have recently attracted substantial attention because of their tiny size (1–1,000 nm) and their capacity to include active chemicals that are either surface-adsorbed or entrapped into the polymeric core. The advantages of utilizing PNPs involved use as carriers for pharmaceuticals due to their ability to release drugs in a controlled manner, their capacity to protect the drugs and other biologically active molecules from the environment, and the alteration of their bioavailability and therapeutic index (Fig. 1B) [59–63]. Depending on their form, PNPs can be either nanospheres or nanocapsules. The polymeric covering on nanocapsules regulates the medication release profile from the core, where it is typically dissolved. Because of their continuous polymeric network, nanospheres can either encapsulate the drug or be adsorbed to its surface [64–66].

PNPs are composed of several synthetic polymers, including poly(g-glutamic acid) [67,68], poly-lactic acid (PLA) [69,70], PLGA [71–75], poly(ethylene glycol) (PEG) [67,76,77], and polystyrene [78,79]. Because of their outstanding biocompatibility and biodegradability, PLA and PLGA NPs have been the subject of most research. However, because of the delayed biodegradation rate, these PNPs either entrap antigen or sustain antigen release for delivery to specific cells. FDA-approved PLGA NPs are highly compatible copolymers comprising PLA and PGA. It has become a popular polymer because it provides various options for long-lasting medication administration. PLGA NPs can encapsulate or adhere the antigen to the surface via covalent or ionic bonding for vaccination applications [74,80]. *Bacillus anthracis*, hepatitis B virus (HBV), ovalbumin, tetanus toxoid (TT), *Plasmodium vivax*, and mono-phosphoryl lipid-A have all been utilized as model antigens to transport antigens in PLGA. Natural polymers, such as chitosan (CS) [81,82], pullulan [83], inulin [84], and alginate [85,86], are also used for NPs preparation because of their biocompatibility and biodegradability, benign makeup, and ease of modification into desirable forms and sizes. In particular, CS-based NPs have been the subject of extensive research [81,82].

Designing with the right polymer is key. Biocompatibility, regulatory approval, and water degradability make PLGA a popular synthetic polymer. This controls antigen release, promoting germinal center responses and immunological activation [29,87]. However, PLGA degradation produces acidic by-products that might damage fragile antigens, requiring formulation changes. Catechol polymers, such as PEI and poly(β-amino esters), benefit nucleic acid vaccinations by forming electrostatic interactions with RNA/DNA and allowing endosomal escape. This improves MHC-I cross-presentation and cytosolic delivery [47,87]. Their positive surface charge helps APCs bind, but it might harm cells; hence, surface shielding, such as PEGylation, is often needed. While PEGylated polymeric systems improve systemic stability, limit nonspecific protein adsorption, and regulate biodistribution, high PEG density may impair cellular uptake and cause immunological responses [76,77]. Bioactivity and biocompatibility are the advantages of natural polymers, but mechanical stability and repeatability are issues. Mucosal vaccine administration with chitosan promotes innate immune activation and epithelial transport. However, its pH-dependent solubility and batch-to-batch variability may impact formulation uniformity [88,89]. Gelatin, dextran, and alginate can encapsulate antigens, whereas synthetic approaches offer greater control over breakdown and release [89]. Synthetic polymers have engineering accuracy, controlled degradation, and regulatory familiarity, while natural polymers are biologically compatible and functionally bioactive. Immunological objective, antigen type, and injection manner affect optimal decision-making. Cationic polymers facilitate the transfer of nucleic acids into the cytosol, accelerate depot-type vaccine administration, and enhance chitosan-based mucosal immunization. PLGA is often the best choice in these situations. The discovered mechanistic variety suggests that immunological outcomes should influence polymer selection, not NP features.

### Lipid-based nanoparticles

The pharmaceutical industry has become interested in liposomes, an early kind of LNPs, as prospective delivery mechanisms for various types of treatments. Because of their importance in properly storing and delivering mRNA to cells, LNPs are now recognized as crucial components of mRNA vaccines for COVID-19, whereas the solid lipid nanoparticles (SLNs), nanostructured lipid carriers (NLCs), and positively charged LNPs–nucleic acid complexes are examples of later generations of lipid nanocarriers with more complicated topologies and physical stabilities (Fig. 1C) [90,91]. Liposomes are a very adaptable nanocarrier system for the administration of nanomedicine; they may transport a diverse array of molecules, containing proteins and nucleic acids, tiny molecules, and hydrophilic or hydrophobic molecules. A wide range of liposomal drugs is currently utilized in hospitals and clinics. Among the many nanomedicine delivery methods that have gone from theory to practice, liposomes pioneered. For instance, Doxil was the first liposomal therapy to receive FDA approval for the treatment of ovarian cancer, an LNP formulation of the anticancer drug doxorubicin [92]. Epaxal (inactivated virus adsorbed on the surface of liposomes) also got the license for hepatitis vaccine delivery [93]. Liposome NPs’ size is between 20 and 1,000 nm, and they contain one or several lipid bilayers; typical substituents comprise stabilizers like cholesterol and phospholipids such as phosphatidylserines (PS), phosphatidylcholine, phosphatidylethanolamine, and phosphatidylglycerols [91]. Many liposomes, such as unilamellar or multilamellar vesicles, have been used in vaccination experiments made of biodegradable phospholipids. Liposomes deliver vaccines by merging with the target cell membrane [58]. Previous studies indicated that expulsing antigenic proteins within multilamellar lipid vesicles provokes robust B- and T-cell responses [94]. To increase T-helper-mediated immune response, APCs readily absorbed similar antigenic peptides encapsulated in PS-liposomes [95].

The structural and functional diversity of lipid-based vaccine nanocarrier platforms is notable. The method of production substantially influences their capacity to cross cell membranes, protect cargo, activate the immune response, and find clinical applications. Conventional liposomes, made up of phospholipid bilayers that integrate hydrophobic and encapsulate hydrophilic substances, exhibit biocompatibility and have been thoroughly investigated in the context of drug and antigen delivery systems. Their bilayer shape alone is insufficient for transporting nucleic acids into cells or out of endosomes, which limits their utility in current mRNA vaccination [91,96]. To enhance controlled release and physical stability, SLNs and second-generation carriers, such as NLCs, were developed using a solid lipid matrix. While the rigid cores of SLNs pose challenges for loading and releasing large, charged biomolecules within cells, they offer superior shelf stability and prolonged release compared to certain liposomal systems [97]. Cationic liposomes enhance the absorption of nucleic acids and facilitate electrostatic complexation due to the presence of permanently charged lipids. These systems enhance cells’ ability to take up nucleic acids; however, their persistently positive surface charge binds more strongly to serum proteins and innate immune components, potentially leading to inflammation and restricting tolerance in vivo [98].

Current mRNA vaccines utilize ionizable LNPs that maintain neutrality at physiological pH but undergo protonation in acidic endosomes. This facilitates endosomal escape and pH-responsive membrane degradation, essential for mRNA delivery to the cytosol and subsequent antigen expression [41,96]. The lipid components of LNPs consist of ionizable lipids, helper phospholipids, cholesterol, and PEGylated lipids. These components play a crucial role in stabilizing particles, facilitating their movement throughout the body, and enabling their release into cells [91]. Mechanistic studies have demonstrated that endosomes protonate ionizable lipids, such as those found in FDA-approved COVID-19 vaccines, thereby aiding mRNA complexation and minimizing systemic toxicity at neutral pH. The endosomal escape of LNP-encapsulated mRNA remains the primary challenge and focus for optimization in ongoing studies [96,99]. Recent studies indicate that lipid composition and tail unsaturation play crucial roles in encapsulation and endosomal release, facilitating in vivo administration [100]. The success of LNPs in clinical trials and vaccine approval can be attributed to their robust mRNA retention, efficient lymphatic and cellular distribution, pH-triggered cytosolic access, and scalable production. This distinguishes them from previous lipid platforms that exhibited low tolerability and ineffective endosomal escape mechanisms [91,98]. The investigation into the immunostimulatory effects of lipid components and innate responses provides valuable insights for advancing next-generation lipid development [98].

### Virus-like particles

VLPs have numerous uses in drug delivery, vaccination, and diagnostics. Many commercially available VLP-based vaccinations are used to prevent infectious diseases, and many more are being developed in clinical trials at various stages of development, thanks to recent developments in biomedical engineering technology. VLP-based techniques might be used more frequently in the future due to their desirable qualities of efficacy, safety, and diversity [101]. The term “virus-like particles” was initially used by researchers to describe tobacco mosaic virus (TMV) particles consisting only of RNA and protein [102]. Most VLPs, composed of multiple copies of monomers arranged in quasi-equivalent conformations to form helical (rod-shaped) or icosahedral structures, are created by proteins when they spontaneously start nucleocapsids. The ultimate shape of VLPs frequently resembles the symmetry of the original parental virus during the assembly stage; however, it might vary depending on the nucleic acid concentration or biophysical conditions [103]. Later, researchers looked more closely at these VLP nanostructures and found that VLPs can stimulate an efficient immune response. The VLP platform can overcome problems seen with traditional vaccinations (like inactivated vaccines and LAV), including inadequate immune response, potential for mutation or toxicity, reversion to harmful forms, extended development timelines, and low production yields (Fig. 1D) [104,105]. Recent developments in mRNA, DNA, and recombinant viral-vector-driven vaccines present effective methods for vaccine production for challenging pathogens and the control of infectious disease epidemics [106]. The size range (20–200 nm) is ideal for allowing them to drain into lymph nodes freely and for easier uptake by APCs, specifically DCs, which will then process and deliver the antigen using MHC class II molecules [107]. By adding pathogen-specific antigens to VLPs, the potent immunogenic effects of VLPs can be used to create vaccines against any pathogen. Technology based on VLPs is progressing concurrently with mRNA and offers an alternative platform for developing efficient vaccines to treat infectious diseases of great concern [108]. Moreover, VLPs have been shown to enhance the immunogenicity of low-potency antigens. For instance, VLPs were also used to represent the antigens, such as the *Salmonella typhi* membrane antigen, influenza A M2 protein, and H1V1 Nef gonadotropin-releasing hormone, all of which generated stronger specific immune responses [109,110].

### Micelles

Micelle is an amphiphilic block copolymer that self-assembles into spherical, colloidal, supramolecular nanostructures (10 to 100 nm) by combining hydrophilic and hydrophobic units. Hydrophobic molecules can be preserved until a drug delivery mechanism facilitates their release, as the core of the micelle exhibits hydrophobic properties. Conventional micelles comprise the hydrocarbon component of long fatty acids, with a hydrophilic polar or charged “head” group and a hydrophobic “tail” group. The sizes of the micelle are measured by the surfactant’s molecular size and other physical characteristics (Fig. 1E) [111–113]. Graft copolymers, tri-block (A-B-A), di-block (A-B), and other amphiphilic copolymers can be used to make micelles. PEG is the most popular hydrophilic building block for di-block and tri-block copolymers. Numerous other corona-forming polymers, such as poly(*N*-isopropyl acrylamide) and poly(*N*-vinyl pyrrolidone), have also been reported. Polycaprolactone, PLGA, poly(propylene oxide), PLA, poly(l-aspartic acid), poly(-amino ester), poly (l-histidine), and phospho-lipid residues such as disteroyl phosphatidyl ethanolamine, short in length and hydrophobic, are just a few of the unique core-forming blocks that have been reported thus far [112].

Micellar NPs have historically been used as drug delivery systems by encasing or protecting hydrophobic pharmaceuticals in the micelle core [84] and have recently been investigated as highly effective adjuvants for the delivery of vaccines. Micellar NPs present distinct benefits compared to non-micellar adjuvant systems, primarily due to their small size (usually around 100 nm), which facilitates the antigen’s access to APCs, such as DCs, within the draining lymph nodes. By traveling directly through lymphatics to lymph nodes, where DCs are concentrated in more numbers than in the periphery, these micelles can target DCs, aiding the development of germinal centers [114]. Depending on the biocompatible hydrophilic micelle corona segments, micellar NPs can also readily exhibit acceptable surface characteristics (nature and surface charge). The surface characteristics of the carrier have been predicted as a crucial factor in the immunological response development [44].

### Immunostimulating complex

The first description of immunostimulating complexes (ISCOMs) by Morein et al. was published in 1984. ISCOM adjuvants comprise phospholipids, cholesterol, and protein antigens, forming a complex. Saponins (Quil-A is extracted from Quillaja Saponaria Molina tree bark, which is found in South America) and cholesterol interact strongly to create a 40-nm size of the unique cage-like particulate structure, which is anticipated to increase the adjuvant’s stability while concurrently lowering the saponins’ hemolytic activity, which is essential for the adjuvant’s safety (Fig. 1F) [115,116]. Through polar interactions, these spherical particles can capture the antigen [80]. The ISCOMs enable the hydrophobic binding of Quillaja saponins into a particular supramolecular structure, permitting the selective integration of viral envelop proteins.

According to the literature, ISCOMs carrying antigens from *Echinococcus granulosus* protoscoles successfully induced mice’s serum antibody responses. Recent research has shown that combining the influenza virus with the B subunit of CT in ISCOMs and administering them through mucosal injection boosts the immune response, resulting in strong mucosal immunoglobulin A (IgA) and systemic immunological reactions [117]. Any antigen with a hydrophobic domain, including proteins found in cell membranes, peptides with hydrophobic domains, and virus envelope proteins, can act as an ISCOM antigen. By structural modifications, such as partial protein denaturation with urea, exposure to acidic conditions, or high temperatures that disclose interior hydrophobic protein regions, non-amphipathic proteins, like HIV gp120, can be integrated into an ISCOM. Another method is to covalently connect soluble proteins with fatty acid tails so that they can be incorporated into ISCOMs or linked to an ISCOM matrix that has already been created [118]. Parenteral administrations are followed by the end of ISCOMs. ISCOMs increase MHC class II expression on APCs [119,120]. The proinflammatory cytokines IL-1 and IL-6 are found in substantial concentrations in mouse spleen and peritoneal cells after in vitro stimulation. In addition, after in vivo activation of mice with influenza ISCOMs, IL-12 can be found in the blood. Tumor necrosis factor α and granulocyte-macrophage colony-stimulating factor (GM-CSF) are likewise inducible by ISCOMs, though less potently [118,121].

### Extracellular vesicles

Almost all types of cells, from bacteria to humans, release EVs, facilitating intercellular communication by transporting heterogeneous substances, i.e., lipids, proteins, RNAs, and DNA. Owing to the existence of the lipid bilayer membrane, cellular DNases, RNases, proteases, and other deteriorating materials are protected more effectively than unenveloped circulating macromolecules like antibodies and cytokines [122,123]. The engineered outer membrane vesicles of gram-negative bacteria (like *Escherichia coli*) are used as a noninfectious nanovaccine development against diverse antigens, which can encourage a higher humoral immune response against the loaded antigen [124]. EVs participate in various biological activities, such as immunological responses. EVs from infected cells can spread viral proteins to immunological and noninfected cells to mask the infection or provoke an answer. Still, viruses can also control EV biogenesis processes by applying themselves [125]. In 1981, Trams et al. [126] used “exosomes” to refer to vesicles released by healthy and cancerous cells with 5′-nucleotidase activities. EVs include exosomes, apoptotic bodies, and microvesicles (sometimes called microparticles or ectosomes). EVs can be divided based on their particle size and cellular origin. Exosomes whose sizes range from 20 to 150 nm are produced through exocytosis from multivesicular bodies (MVBs). In contrast, microvesicles, with sizes between 100 and 1,000 nm, are released directly from plasma membranes through budding or shedding. Dying cells expel apoptotic bodies (50 to 5,000 nm) (Fig. 1G) [127–130]. The production of EVs by nearly all live cells has been proven, and EVs have also been isolated from the majority of physiological fluids, including urine, saliva [131], plasma [132], and breast milk [133]. Exosomes produce specific proteins, such as Alix, TSG101, and clathrin, that are involved in the synthesis of MVBs and the endocytosis of the plasma membrane. They also express tetraspanins, or CD63, CD9, and CD81, which are involved in membrane trafficking, as well as Ras-associated binding proteins and annexins. Researchers can easily distinguish exosomes from other nonclassical exosomes that lack exosomal markers like CD63, CD9, and CD81, thanks to the enrichment of these proteins [134].

Interestingly, the NPs described above could be carefully chosen for specific immunization goals, such as delivering to the cytosol to activate CTLs via MHC-I cross-presentation or providing sustained release to generate strong antibody responses through extended antigen exposure. The surface groups of inorganic NPs can be exploited to deliver siRNA/DNA into the cytosol with precision and to activate CTLs [135], whereas for CTL activation, LNPs (liposomes) enable endosomal escape and membrane fusion for mRNA/protein access, while micelles (~100 nm) efficiently deliver peptides, promoting lymphatic DC targeting [41,136]. Also, the VLPs (20 to 200 nm size) facilitate DC uptake with endosome-to-cytosol trafficking [137].

Conversely, for sustained antigen release, PNPs such as PLGA/PLA excel due to their delayed biodegradation, which creates depots for humoral immunity [138]. The ISCOMs (40 nm cages) sustain cytokine induction (IL-6/IL-12) and support serum/mucosal IgA, supporting the sustained antigen release [117,118,121]. Therefore, optimized nanovaccine design for precise immunity profiles can be achieved by using cytosolic-focused NPs to elicit CD8^+^ responses or by employing PLGA/ISCOMs to sustain IgG/IgA responses.

In summary, the studies above signify the transformative potential of NP-based platforms for nanovaccine design. Inorganic NPs, such as gold, silica, and aluminum, exhibit excellent synthetic precision and adjuvant potency but are limited by their nonbiodegradability and potential for accumulation. Instead, the polymer-based nanoplatforms, PLGA and chitosan, offer superior biocompatibility, sustained antigen release, and protection. However, they depict slower degradation profiles. Furthermore, the LNPs, including liposomes and mRNA-carrying LNPs, demonstrate remarkable versatility in delivering diverse cargos via membrane fusion, though systemic stability remains a challenge; meanwhile, VLPs emerge as a major candidate, offering nonreplicative safety, viral mimicry for enhanced APC uptake, and lymph node drainage. These VLPs have an enhanced ability to amplify weak antigens in comparison to conventional vaccines. On the other hand, micelles provide agile lymphatic targeting for DC activation, whereas ISCOMs deliver cytokine-driven mucosal immunity via saponin structures, which are ideal for hydrophobic antigens outpacing the natural shielding of EVs despite their scalability issues. Eventually, the VLPs and LNPs are nanoplatforms that stand out for their safety, adaptability, and translational promise for the development of nanovaccines, positioning them as cornerstones for combating infectious diseases, with innovations that will refine their clinical efficacy and global impact in the future.

## NP-Based Antigen Delivery for Immunization

Currently, NPs are employed in the treatment of several infectious illnesses. The primary focus of NP-based vaccination research is on 3 categories of diseases: viral infections, bacterial infections, and cancer. NP-based vaccines employ meticulously engineered NPs to augment immune responses to viral infections. These vaccines enhance antigen delivery, stability, and immunogenicity by emulating viral architecture. Diverse NPs, encompassing lipid and polymer-based carriers, enhance immunizations for diseases such as hepatitis B, influenza, and HIV. A CS-NP-based nasal vaccine for HBV exhibited an evolving immune response in mice, but M2e-AuNP-based formulations conferred enduring protection against influenza. Moreover, self-amplifying RNA (saRNA) vaccines encapsulated in LNPs have been developed, enabling fast responses during pandemics, as demonstrated by vaccinations targeting SARS-CoV-2. Research demonstrates that nanoparticulate vaccines can improve immune responses against bacterial infections, with effective formulations aimed at pathogens such as *Burkholderia mallei* and *B. anthracis*. These sophisticated vaccines demonstrate the promise of NP technology in vaccination techniques. Table 1 lists nanovaccines that were in clinical trials.

**Table 1.** List of nanovaccines in clinical trials

Clinical Trials.gov ID	Trial phase	Types of nanovaccine	Study title	Conditions	Sponsor
NCT01704365	Phase II (completed)	RSV-F protein nanoparticle vaccine	RSV-F Vaccine Dose-Ranging Study in Young Women	Respiratory syncytial virus	Novavax
NCT04935801	Phase I (completed)	Gold nanoparticle-based peptide vaccine	A Phase-I Study of a Nanoparticle-based Peptide Vaccine Against Dengue Virus	Dengue virus	Emergex Vaccines Holding Ltd.
NCT02247726	Phase II (completed)	RSV-F protein nanoparticle vaccine	RSV F Vaccine Maternal Immunization Study in Healthy Third-Trimester Pregnant Women	Respiratory syncytial virus	Novavax
NCT01960686	Phase II (completed)	RSV-F protein nanoparticle vaccine	RSV F Dose-Ranging Study in Women	Respiratory syncytial virus	Novavax
NCT01709019	Phase I (completed)	RSV-F protein nanoparticle vaccine with licensed inactivated influenza vaccine	RSV-F Vaccine and Influenza Vaccine Co-Administration Study in the Elderly	Respiratory syncytial virus	Novavax
NCT02370589	Phase 1 (completed)	Ebola virus (EBOV) glycoprotein (GP) nanoparticle vaccine	Study to Evaluate the Immunogenicity and Safety of an Ebola Virus (EBOV) Glycoprotein (GP) Vaccine in Healthy Subjects	Ebola virus	Novavax
NCT05639894	Phase I/IIa	RSV vaccine encapsulated with LNPs	Study of a Respiratory Syncytial Virus Candidate Encapsulated in a Lipid Nanoparticle-Based Formulation in Adults Aged 18 to 50 Years and 60 Years and Older	Respiratory syncytial virus	Sanofi Pasteur, a Sanofi Company
NCT02296463	Phase I (completed)	Respiratory syncytial virus (RSV) recombinant fusion (F) nanoparticle vaccine	A Phase I Randomized, Observer-Blinded, Dose-Ranging Study in Healthy Subjects 24 to <72 Months of Age	Respiratory syncytial virus	Novavax
NCT05113862	Phase I (completed)	T-cell priming specific cocktail of coronaviruses peptides mounted on a gold nanoparticle	A Phase-I Study of a Nanoparticle-Based Peptide Vaccine against SARS-CoV-2	Coronavirus; SARS-CoV-2 infection; COVID-19	Emergex Vaccines Holding Ltd.
NCT02593071	Phase II (completed)	Respiratory syncytial virus (RSV) recombinant F nanoparticle vaccine	Safety and Immunogenicity of the RSV-F Vaccine in Older Adults Previously Treated with the Same Vaccine or Placebo in the Prior Year	Respiratory syncytial virus	Novavax
NCT05519839	Phase II (completed)	SARS-CoV-2 recombinant spike (rS) (SARS-CoV-2 rS) nanoparticle and quadrivalent hemagglutinin (HA) nanoparticle influenza vaccine	A Study to Evaluate the Safety and Immunogenicity of COVID-19 and Influenza Combination Vaccine (COVID-19)	COVID-19; influenza	Novavax
NCT05542693	Phase II	Lipid-inorganic nanoparticle (LION) formulated replicating RNA-based vaccine	Safety and Immunogenicity of the RNA MCTI CIMATEC HDT Vaccine	COVID-19; COVID-19 vaccine	Azidus Brasil
NCT05633446	Phase I–II	PepGNP-COVID19	Next Generation T-cell Vaccine against Coronavirus Disease (COVID-19)	Coronavirus; SARS-CoV-2 infection; COVID-19	Emergex Vaccines Holding Ltd.
NCT03293498	Phase I–II (completed)	Recombinant trivalent nanoparticle influenza vaccine	Evaluation of the Safety and Immunogenicity of a Recombinant Trivalent Nanoparticle Influenza Vaccine with Matrix M-1 Adjuvant (NanoFlu)	Influenza	Novavax
NCT05925127	Phase II–III	SARS-CoV-2 recombinant (r) spike (S) protein nanoparticle (SARS-CoV-2 rS) vaccines	Phase 2/3 Heterologous Boosting Study with Different Dose Levels of Monovalent SARS-CoV-2 rS Vaccines (COVID-19)	COVID-19	Novavax
NCT05372588	Phase III (completed)	SARS-CoV-2 rS adjuvanted with Matrix-M adjuvant and bivalent (NVX-CoV2373 [prototype] + Omicron subvariant) SARS-CoV-2 rS vaccines	Phase 3 Boosting Study for the SARS-CoV-2 rS Variant Vaccines (COVID-19)	COVID-19; SARS CoV 2 Infection	Novavax
NCT05876364	Phase I	Lipid-inorganic nanoparticle (LION) formulated replicating RNA-based vaccine	Study to Assess Safety, Reactogenicity and Immunogenicity of the repRNA (QTP104) Vaccine against SARS-CoV-2 (COVID-19)	COVID-19; SARS-CoV-2	Quratis Inc.
NCT04120194	Phase III (completed)	Recombinant quadrivalent nanoparticle influenza vaccine (Quad-NIV)	Phase 3 Pivotal Trial of NanoFlu in Older Adults	Influenza Human	Novavax
NCT05658523	Phase III (completed)	Stabilized S protein of SARS-CoV-2 formulated in RNA-LNPs	COVID-19 Booster Study in Healthy Adults in Australia	COVID-19	Murdoch Children’s Research Institute
NCT04776317	Phase I (completed)	Self-amplifying mRNA–LNPs–Spike (SAM-LNP-S	Chimpanzee Adenovirus and Self-Amplifying mRNA Prime-Boost Prophylactic Vaccines against SARS-CoV-2 in Healthy Adults	COVID-19	National Institute of Allergy and Infectious Diseases (NIAID)
NCT05515042	Phase II	SARS-CoV-2 rS (CovovaxTM) recombinant protein nanoparticle vaccine	Phase 2a Trial to Evaluate Safety and Immunogenicity of COVID-19 Vaccine Strategies in HIV-infected/Uninfected Adults (AUR1-8-341)	COVID-19	The Aurum Institute NPC
NCT0620552	Phase I	LNP-encapsulated circRNA-based vaccine	A Study to Evaluate Safety and Immunogenicity of TI-0010 SARS-CoV-2 Vaccine in Healthy Adults	COVID-19 immunization	National Drug Clinical Trial Institute of the Second Affiliated Hospital of Bengbu Medical College
NCT04533399	Phase II (completed)	SARS-CoV-2 recombinant spike protein nanoparticle vaccine	A Study Looking at the Effectiveness and Safety of a COVID-19 Vaccine in South African Adults	SARS-CoV-2 infection, COVID-19	Novavax
NCT05516459	Observational (patient registry)	Spike protein of SARS-CoV-2, which is encapsulated in LNPs	Prospective Monitoring of BNT162b2 Second Vaccination Booster Effects in Health Care Workers (HCW)	Coronavirus infections	Soroka University Medical Center
NCT04816669	Phase III (completed)	Frozen-liquid BNT162b2 with lipid nanoparticle	A Study to Evaluate Safety, Tolerability, & Immunogenicity of Multiple Formulations of BNT162b2 against COVID-19 in Healthy Adults	SARS-CoV-2 infection; COVID-19	BioNTech SE
NCT04785144	Phase I (completed)	Lipid nanoparticle (LNP)-encapsulated mRNA-based vaccine	Safety and Immunogenicity Study of a SARS-CoV-2 (COVID-19) Variant Vaccine (mRNA-1273.351) in Naïve and Previously Vaccinated Adults	COVID-19; COVID-19 immunization	National Institute of Allergy and Infectious Diseases (NIAID)
NT05057169	Phase IV	BNT162b2, nucleoside-modified mRNA encoding the trimerized SARS-CoV-2 spike glycoprotein in LNPs	Randomized Trial of COVID-19 Booster Vaccinations (Cobovax Study)	COVID-19 vaccination	The University of Hong Kong
NCT05945485	Phase I	mRNA vaccine encoding the full length HA of influenza A/California/07/2009 (H1N1) encapsulated in lipid nanoparticle	A Study to Evaluate the Safety and Immunogenicity of Two Doses of DCVC H1 HA mRNA-LNP in Healthy Adults	Influenza	National Institute of Allergy and Infectious Diseases (NIAID)
NCT06069544	Phase I/IIa	RNA-based vaccine (BNT165e) encapsulated in LNPs	A Clinical Trial to Evaluate the Safety, Efficacy and Immune Responses after Vaccination with an Investigational RNA-based Vaccine against Malaria	Malaria	BioNTech SE
NCT05581641	Phase I	BNT165b1, a ribonucleic acid (RNA)-lipid nanoparticle (LNP) encoding for part of the *Plasmodium falciparum* circumsporozoite protein (PfCSP)	Safety and Immune Responses after Vaccination with an Investigational RNA-based Vaccine against Malaria	Malaria	BioNTech SE

### NP-based vaccine against viral infections

NP-based vaccinations utilize specifically designed NPs, such LNPs, protein-based carriers, or polymer-based carriers, to administer antigens and enhance immune responses to viral infections. These vaccines enhance immune system activation, optimize targeted delivery, and increase antigen stability. They enhance immunogenicity and provide more robust, long-lasting protection by simulating viral structures. This includes mRNA vaccines, commonly referred to as COVID-19 vaccines, and VLP immunizations. Various forms of inorganic and organic NPs are utilized to develop vaccines for viral infections. Table 2 enlists certain nanovaccines against viral infections approved for human use.

**Table 2.** List of approved nanovaccines against viral infection

Vaccine name	Developer	Target disease	Type of nanoparticles	Antigens type	Year of authorization
BNT162b2	Pfizer-BioNTech	SARS-CoV-2	LNPs	Spike glycoprotein mRNA	2020 by FDA, MHRA, and EMA
mRNA-1273	Moderna	SARS-CoV-2	LNPs	Spike glycoprotein mRNA	2020 by FDA, MHRA, and EMA
Shingrix	GlaxoSmithKline	Herpes Zoster	Liposomes	Recombinant glycoprotein E	2017 by FDA
Gardasil-9	Merck	Human papillomavirus types 6, 11, 16, 18, 31, 33, 45, 52, and 58	VLPs	Recombinant L1 proteins	2015 by EMA
Cervarix	GlaxoSmithKline	Human papillomavirus types 16 and 18	VLPs	Recombinant L1 proteins	2007 by EMA
Gardasil	Merck	Human papillomavirus types 6, 11, 16, and 18	VLPs	Recombinant L1 proteins	2006 by EMA
Engerix-B	GlaxoSmithKline	Hepatitis B	VLPs	Recombinant HBsAg	2000 by EMA
Inflexal V	Crucell, Berna Biotech	Influenza H1N1, H3N2, and B	VLPs	Hemagglutinin and neuraminidase	1997 by Switzerland, UK, and EU country
Epaxal	Crucell, Berna Biotech	Hepatitis A	Liposomes	Inactivated HAV	1993 by EMA
Recombivax HB	Merk	Hepatitis B	VLPs	Recombinant HBsAg	1986 by FDA

**Fig. 2.**
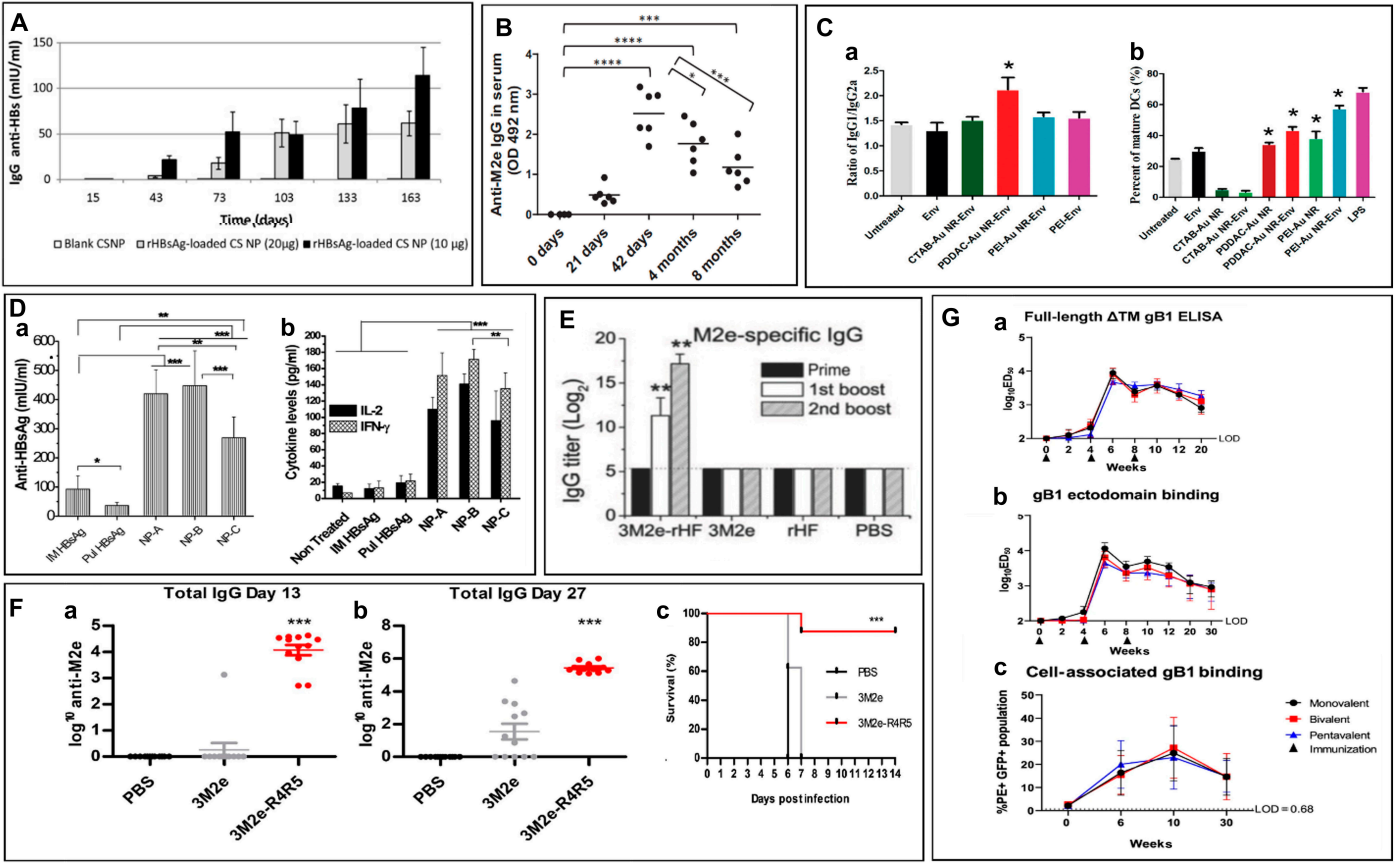
Immune response of NP-based antigen delivery against viral infection. (A) Serum IgG antibody levels after intranasal administration of rHBsAg-loaded CS-based NPs at 2 different doses (10 and 20 μg) as well as blank NPs. All groups received 2 doses: at time 0 and after 28 days. Reprinted with permission from Prego et al. [139]. Copyright (2010) Elsevier. (B) Mice (*n* = 6) were vaccinated with 500 M2e-AuNP + sCpG on days 0 and 21, and serum was collected on day 0, day 21, day 42, 4 months, and 8 months for analysis. M2e-specific IgG antibody in 1:400 diluted serum of individual mice. Each circle represents an animal, and the horizontal bar represents the mean. Reprinted with permission from Tao and Gill [52]. Copyright (2015) Elsevier. (C) The effects of Au NRs on the immune response and dendritic cell maturation. (a) Determination of the type of immune response after mice immunized with the Au NR–Env plasmid DNA complex. (b) The effect of Au NRs and the Au NR–Env complex on DC maturation. Reprinted with permission from Xu et al. [142]. Copyright (2012) American Chemical Society. (D) (a) Immune response profiles of nanoparticulate based formulations at the end of 28 days after dosing of NP-A (PLA based, 943 nm), NP-B (PLGA 85:15 based, 774 nm), and NP-C (PLGA 50:50 based, 474 nm) formulations on days 0 and 14. Data represent mean ± SD, *n* = 6 to 9. (b) Interferon-γ (IFN-γ) and interleukin-2 (IL-2) levels in spleen homogenates of rats immunized with different formulations and control groups. IFN-γ and IL-2 levels were assayed 42 days after the first dose of the formulations. Data represent mean ± SD, *n* = 3 to 8 (*results are significantly different, *P* < 0.05; **results are significantly different, *P* < 0.01; ***results are significantly different, *P* < 0.001). Reprinted with permission from Thomas et al. [143]. Copyright (2011) American Chemical Society. (E) M2e-specific IgG titers in sera collected 2 weeks after each immunization, *n* = 6. Reprinted with permission from Qi et al. [144]. Copyright (2018) WILEY-VCH Verlag GmbH & Co. KGaA, Weinheim. (F) Intranasal delivery of 3M2e-R4R5 nanovaccine induces a strong local and systemic humoral response. (a and b) Total IgG titers from mice serum after the (a) first and (b) second intranasal immunization. (c) Survival of immunized mice upon infection with 5 × LD_50_ of influenza A/Puerto Rico/8/1934. Log rank Mantel–Cox statistical test was used; *P < 0.05, **P < 0.01, *** *P* < 0.001. M2e-specific IgG titers in sera collected 2 weeks after each immunization, *n* = 6. Reprinted with permission from St-Louis et al. [145]. Copyright (2024) Elsevier. (G) The dynamics of rabbit plasma IgG binding magnitude to soluble full-length gB-1 with the deletion of transmembrane domain (a) and soluble gB-1 ectodomain (b) was measured by ELISA, and the IgG binding to cell-associated full-length gB-1 (c) for the preimmune (week 0), peak immunogenicity (weeks 6 and 10), and durability (week 30) time points was estimated by gB-transfected cell binding assay. Data points are shown as the average IgG binding response with one standard deviation. A statistically significant difference was observed in the binding to the soluble gB-1 ectodomain (*P* value 0.002, FDR-adjusted *P* value of 0.006). This figure is reproduced under the terms of the Creative Commons CC BY license. Copyright (2024) Nature [147].

The efficacy of CS-NP-based nasal vaccine against HBV was evaluated utilizing a boost-dose schedule with recombinant hepatitis B surface antigen (rHBsAg) and adjuvants after intranasal immunization at 10- and 20-μg dosages, with blank NPs as a control. Regardless of quantity, anti-rHBsAg IgG levels in mice demonstrated a modest but developing immune response over time. Even though vaccine recipients are referred to as “low responders”, antibody concentrations in both cases were around 10 and 100 U/ml, which is thought to be seroprotective against hepatitis B. As anticipated, blank NPs did not cause any particular IgG response (Fig. 2A) [139]. Mice were given intranasal delivery of CS NPs coated in alginate and contained rHBsAg. Analyzing the adjuvant impact of the delivery method involved measuring interferon (IFN)-c production in spleen cell supernatants, anti-HBsAg IgG levels in the blood, anti-HBsAg, secretory-IgA (sIgA) levels in vaginal and nasal secretions, or feces extracts [140]. Newcastle disease virus (NDV) pathogenic strain is a highly contagious virus that primarily infects poultry, causing Newcastle disease. Live NDV vaccinations were given via eye drops, a coarse aerosol spray, or drinking water. However, this vaccine could not produce enough mucosal immune response. To overcome the shortcomings, the CS-NPs were used as an antigen carrier. CS-NPs carried live and inactivated NDV virus for vaccination and produced enough mucosal immune response [141].

The findings demonstrate that a cross-protective influenza vaccine, incorporating M2e immobilized on AuNPs along with soluble Cytosine-phosphorothioate-Guanine (CpG) as an adjuvant, can effectively protect against various influenza A subtypes, thereby reducing the chances of seasonal epidemics and pandemics. The M2 membrane protein’s (M2e) extracellular domain immobilized on AuNPs can produce protective immunogenicity against multiple influenza A subtypes when combined with soluble CpG as an adjuvant. The vaccine formulation comprises soluble M2e, which generates greater anti-M2e antibodies and safeguards female BALB/c mice against deadly infections caused by the influenza virus. The 500M2e-AuNP+sCpG vaccine formulation produced lifetime immunity and provided complete defense against illness and mortality following the challenge of influenza virus A/PR8/34 (H1N1) 8 months after the initial immunization. In free M2e vaccinated mice, a gradual decline in serum anti-M2e antibodies was observed. The successful results show that M2e-AuNP+sCpG could be used as a worldwide influenza A vaccine (Fig. 2B) [52]. Similarly, surface-engineered gold nanorods (AuNRs) are being explored as a potential DNA vaccine adjuvant for HIV therapy. The impact of surface chemistry on the adjuvant efficacy of the AuNR has been explored through the application of 3 distinct molecules—cetyltrimethylammonium bromide, poly(diallydimethylammonium chloride) (PDDAC), and polyethyleneimine (PEI)—to the surface of the nanorod. AuNRs with novel surface engineering are being tested as a potential DNA adjuvant for HIV treatment. HIV Env plasmid DNA carried by PDDAC- and PEI-AuNRs may provide prospective candidate adjuvants for therapeutic usage by markedly boosting both cellular and humoral immunity, as well as T-cell proliferation, and directly stimulating DC maturation, unlike the administration of untreated HIV-1 Env plasmid DNA in vivo (Fig. 2C) [142].

Porous PLA and PLGA NPs were assessed for their potential in the pulmonary delivery of the hepatitis B vaccine. A double-emulsion solvent-evaporation procedure added a specific amount of HBsAg to PLGA and PLA NPs. After the intramuscular delivery of simple HBsAg, the saliva showed barely any sIgA response. Compared to both, sIgA levels slightly increased when ordinary HBsAg was given through the pulmonary route. After oral dosing of simple HBsAg, the sIgA response in saliva was insignificant. Contrary to the simple intramuscular or pulmonary administration of HBsAg, the nanoparticulate formulations of PLA and PLGA with varying sizes when immunized pulmonarily exhibited a notable enhancement in the HBsAg-specific immune response and substantially increased the secretion of IL-2 and IFN-γ cytokine level after 28 days in female Sprague−Dawley rats (Fig. 2D) [143].

Even without an adjuvant, intranasal administration of the self-assembling recombinant human heavy chain ferritin (rHF) cage with 3M2e (3M2e-rHF) stimulates robust immunological responses in mice. Some of these reactions are high serum M2e-specific IgG antibodies, T cell-based immunological responses, and mucosal sIgA antibodies. The 3M2e-rHF NPs also protect against lethal H1N1 and H9N2 virus infections (Fig. 2E) [144]. Curli-specific gene A (CsgA) is a protein that helps in bacterial biofilm formation. CsgA protein has the properties of self-assembling and formation of monofilaments. The 3 repeats of influenza A virus Me2 protein conjugated with the N-terminus of the 4th and 5th repeating units (R4R5) of the CsgA protein and a protein-based self-adjuvant nanovaccine prepared against influenza virus. The 3M2e-R4R5 nanovaccine formulation was uptaken by APCs (such as DCs) and produced a high humoral immune response in BALB/c mice, showing zero mortality in influenza A (H1N1/Puerto Rico/8/1934) virus-challenged (Fig. 2F) [145]. mRNA vaccines offer a rapid and effective approach for developing universal influenza B vaccinations. One of the studies focused on developing a pentavalent vaccine incorporating all assessed antigens, providing defense against historical and modern influenza B viruses. The vaccination, administered at a low dose of 50 ng per antigen, was crucial in reducing influenza-related morbidity and mortality. Nucleoside-modified mRNA-loaded lipid nanovaccine was prepared as a universal vaccine against the influenza B virus. This vaccine induced immunity against both ancestral and modern influenza B viruses and protected them from both antigenic lineages [146]. Human cytomegalovirus (HCMV) is a prevalent prenatal infection among people with compromised immune systems. The HCMV glycoprotein B subunit (gB) vaccine, adjuvanted with MF59, demonstrated the highest performance among HCMV vaccines, achieving 50% effectiveness against primary HCMV infection. Nonetheless, a study revealed that diminished efficacy was attributed to strain-specific immunity. The research employed monovalent (gB-1), bivalent (gB-1+gB-3), or pentavalent (gB-1+gB-2+gB-3+gB-4+gB-5) gB LNP-encapsulated nucleoside-modified RNA vaccines to immunize 18 female rabbits. In comparison to the monovalent vaccine, the multivalent vaccines did not demonstrate an increased amplitude or breadth of IgG response to the gB ectodomain or cell-associated gB. At peak immunogenicity, multivalent vaccine-induced T-cell responses specific to gB-2 from peripheral blood mononuclear cells were greater than those observed in monovalently vaccinated subjects against a vaccine-mismatched gB genotype. To enhance immunization efficacy, it is essential to understand how to expand the protective scope of the HCMV vaccine (Fig. 2G) [147].

Recombinant Norwalk virus VLPs (rNV VLPs) that cannot replicate are immunogenic when given orally without a delivery mechanism or mucosal adjuvant. rNV VLPs are a good model for studying oral antigen transport and could be used as a mucosal vaccination to prevent NV infections. When given orally to healthy adults without an adjuvant, rNV VLPs are safe and immunogenic and cause reactions in serum IgG and intestinal IgA [148,149]. The influenza virus is a highly contagious respiratory disease that results in severe morbidity and mortality. Infected hen eggs are utilized to cultivate the virus in allantoic fluid for the current trivalent vaccines in the United States. The virus is thereafter chemically inactivated and disassembled into its constituent parts. These vaccinations generate antibodies, mostly targeting the viral hemagglutinin (HA), which are effective for healthy people but less so for high-risk populations such as the elderly and individuals with compromised immune systems. A new influenza VLP has been created as a potential immunization against influenza infection, utilizing a nonegg or nonmammalian cell culture approach to address existing limitations. Following infection with baculovirus vectors that generate an expression cassette including 3 structural proteins of the influenza virus—HA, neuraminidase (NA), and matrix (M)—VLPs were extracted from the supernatants of *Spodoptera frugiperda* Sf9 insect cells. The ferret and mouse immunological response was induced by intramuscular injections of VLPs, varying in HA concentration (3 to 24 ng). All vaccinated animals demonstrated elevated titers of anti-HA antibodies, with those receiving the highest doses of VLPs additionally displaying antibodies against NA. In comparison to rHA or whole inactivated virus (WIV), VLPs induced Th1 immune responses in mice, and purified rHA produced IgG1 antibodies (IgG2a and IgG2b), representing a T helper type 2 (Th2) response capable of recognizing a broader array of antigenically diverse H3N2 virus isolates [150]. VLPs of the influenza virus A/PR8/34 (H1N1)’s HA, matrix (M1), and M2e proteins were tested for immunogenicity, long-term cross-protection, and lung proinflammatory cytokines in mice, of which the intranasal VLPs expressing HA produced potent serum and mucosal antibody titers that had neutralizing action against A/WSN/33 (H1N1), PR8, and A/swine/Iowa/15/30 (H1N1) (1,918 antigenically related influenza virus). VLPs containing HA reduced pulmonary cytokines and protected mice from mouse-adapted WSN or PR8 viruses for 5 months [151–153]. A single- or two-dose regimen of a noninfectious recombinant VLP vaccination based on rHA provided protection in mice and ferrets against influenza A H5N1 clade 1 and clade 2 isolates with pandemic potential [154,155]. HBV core particles, composed of 120 amino acids located at the amino terminus of the nucleocapsid (N) protein, exhibit strong immunogenicity in mice when coupled with adjuvants. This is exemplified by hantaviruses such as Hantaan, Dobrava, or Puumala. HBc-based VLPs elicited robust and targeted antibody responses against the N protein in C57BL/6 and BALB/c mice, even without the use of adjuvant. All IgG subclasses were induced N-specifically [156]. Because of its higher risk, HPV, comprising over 130 epitheliotropic genotypes, is a substantial health concern in underdeveloped countries. Despite the existence of 2 approved preventive vaccines, their prohibitive cost hinders widespread adoption. The production of recombinant immunogens in plants represents a viable strategy for developing effective and cost-efficient vaccines. The capacity of specific HPV genes, such as L1, to autonomously assemble into VLPs renders a plant-derived vaccine, functioning as both a prophylactic and therapeutic alternative, an enticing possibility. The synthesis of a chimeric particle in plants was made from HPV-16 L1-based VLPs containing a string of T-cell epitopes from E6 and E7 attached to the C-terminus. In vivo experiments are underway to see if the particles can induce disease regression and resolve viral infections in C57BL/6 mice, as they triggered a higher antibody and CTLs response. Chimeric particles may be utilized in the creation of therapeutic and preventive vaccines, thereby lowering production expenses [157]. Rotavirus (RV) VLP rectal vaccine protects BALB/c mice from RV. Mice had higher serum and feces anti-RV IgA and IgG titers [158]. Likewise, double-shelled VLPs of the bluetongue virus (BTV) serotypes 1, 2, 10, 13, or 17 are found in recombinant baculoviruses. After purification, either single VLP types (BTV 10 or 17) or all 5 serotypes were mixed and used to immunize sheep. A robust immune response was brought on by double doses of 10 μg of VLPs, which shielded the sheep from the challenge of the homologous pathogenic virus [159]. Potential candidate immunogens for an HIV-1 vaccine are noncontagious Pr55gag VLPs with a substantial amount of oligomeric HIV-1 envelope (Env) proteins [160,161]. After intraperitoneal (i.p.) administration of the HIV-1 clade A isolates (HIV-VLPAs), BALB/c mice developed neutralizing antibodies and CTLs. The intraperitoneal and intranasal injection induced systemic (intestinal) and mucosal (vaginal) IgA and IgG responses. HIV-VLPAs administered intranasally increased CTL activation but were less efficient than intraperitoneal doses. Thus, the HIV-VLPAs boost both arms of immunity and induce specific humoral immunity in mucosal locations, the main entry point for HIV-1 infection, which is of great interest [162]. VLPs are effective HIV-1 potential vaccines. Recent studies show that HIV Env-containing VLPs cause local and distal mucosal immune responses. HIV virions or VLPs have a small amount of Env protein compared to most enveloped viruses. The factors that affect the assimilation of HIV Env particles were analyzed, and a collection of chimeric genes that swapped out the cytoplasmic tail (CT), transmembrane (TM), and signal peptide (SP) domains of HIV-1 Env with those from other cellular or viral proteins was generated. All constructs studied were generated from HIV-1 Con-S CFI gp145, which was more efficiently integrated into VLPs than full-length Env. The chimeric VLPs with increased Env inclusion could be an effective, safe AIDS vaccine immunogen [163].

SARS-CoV-19, a new coronavirus disease, killed approximately 6.69 million people [164]. A self-assembling polypeptide NP exhibits a SARS B-cell epitope derived from the C-terminal heptad repeat of the virus’s spike protein. Biophysical analyses validated the design, demonstrating α-helical NPs measuring 25 nm in size. Immunization trials were conducted using BALB/c mice devoid of adjuvants. The resulting antibodies exhibited remarkable conformation selectivity for the coiled-coil epitope and were capable of identifying the C-terminal heptad repeat region’s native trimeric conformation. Thus, the antisera neutralized an in vitro infection inhibition experiment. SARS-CoV-19 and other enveloped viruses have neutralizing epitopes with coiled-coil shapes, making peptide NPs a suitable vaccination platform [165]. To create a potent, secure, and adaptable nanovaccine, the S1 component (surface protein) of the SARS-CoV-19 virus was combined with amphiphilic adjuvant monophosphoryl lipid A for Toll-like receptor 4 (TLR4) and CpG oligodeoxynucleotide for receptor 9. In BALB/c mice, the nanovaccine triggered humoral immunity and potent IgA antibodies. The serum of vaccinated animals strongly inhibits SARS-CoV-2-infected Vero cells. The nanovaccine activates CD4^+^ and CD8^+^ cells to induce high T-cell immunity compared to free S1 in an Alum adjuvant [166]. saRNA encodes alphaviral replicase and a gene of interest, enabling RNA replication upon entry into the cytoplasm. This approach is highly crucial for vaccine development during a global pandemic, as any required antigen may be encoded using saRNA encapsulated in LNPs. Plasmid-based surface spike protein self-amplifying mRNA (saRNA) loaded LNP-based vaccine prepared against SARS-CoV-2 virus [167–169]. LNP-based recombinant hexapro spike protein-based plasmid DNA vaccine was prepared and immunized in C57BL/6 female mice, resulting in a higher immune response against SARS-CoV-2 variants of concern. The LNP-based pDNA vaccine neutralizes Omicron variants (B.1.1.529) in vivo [170]. VLP-conjugated recombinant S protein-based vaccine also protects hamster models from SARS-CoV-1-like and SARS-CoV-2-like sarbecoviruses [171]. Furthermore, hybridizing bioengineered viral cell membranes with bacterial vesicles creates envelope virus-mimetic nanovaccines called hybrid membrane vesicles (HMVs). HMVs were prepared in the fusion of HEK-293T cells, expressing recombinant SARS-CoV-2 spike proteins in the surfaces of membrane vesicles with the fusion of *Salmonella* bacteria outer membranes. The size of HMVs on dynamic light scattering analysis was 165 ± 2.3 nm with a charge of −11 mV. The HMV uptake by DCs in vitro and in vivo activated humoral and cell-mediated immune response against SARS-CoV-2 virus [172].

### NP-based vaccine against bacterial infection

**Fig. 3.**
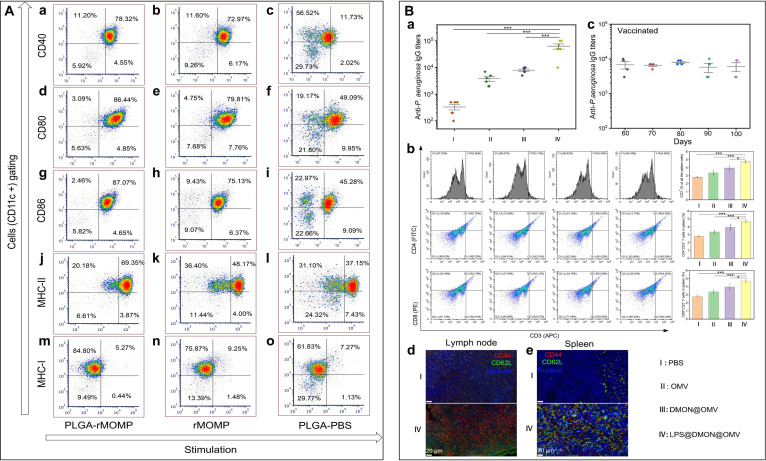
(A) Enhanced expression of co-stimulatory and MHC molecules. DCs were stimulated with 0.01 μg of PLGA-rMOMP or bare rMOMP for 24 h and stained with fluorochrome-conjugated antibodies. Stained cells were subjected to flow cytometric analysis of surface molecules by gating on CD11c^+^ cells, CD40 (a to c), CD80 (d to f), CD86 (g to i), MHC-II (j to l), and MHC-I (m to o). PLGA-PBS was used as a negative control. The upper right quadrants are % positive cells for the respective expression of surface molecules. Experiments were repeated at least 3 times. Reprinted with permission from Sahu et al*.* [177]. Copyright (2020) Elsevier. (B) Nanovaccines induce strong bacteria-specific immune responses in vivo. (a) Anti-*P. aeruginosa* IgG titers at day 21. (b) A representative flow cytometry analysis and histogram of percentage of CD3^+^, CD4^+^CD3^+^, and CD8^+^CD3^+^ T cells in the spleen. (c) Time course of anti-*P. aeruginosa* IgG titers in vaccinated mice. (d) Immunofluorescence staining of CD44 (Red), CD62L (Green), and CD44^+^CD62L^+^, which represented the central memory T cells in inguinal lymph nodes tissue slice. Scale bars, 20 μm. (e) Immunofluorescence staining of CD44^+^CD62L^+^ (central memory T cells) in the spleen. Scale bars, 20 μm. I: PBS; II: OMV; III: DMON@OMV, IV: LPS@DMON@OMV. Data were expressed in the form of mean ± SD (*n* = 6) (ns: not significant; *P < 0.05; **P < 0.01; ***P < 0.001). Reprinted with permission from Wu et al. [180]. Copyright (2022) Elsevier*.*

Like viruses, scientists also use NPs to develop vaccines against bacterial infection. AuNP-based recombinant vaccine against *B. mallei* (Bm) in vivo protects mice. Surface-modified AuNPs conjugated with recombinant (HA, OpcP, and OmpW) proteins, as well as lipopolysaccharide (LPS), were administered intranasally to immunize mice. This nano-glycoconjugate vaccine showed a particular antigen-specific humoral immune response and activated CD8^+^ T cells and memory T cells in mice [173]. Biological weapon *B. anthracis* causes anthrax. Vaccination is the safest anthrax prevention method. Current anthrax vaccines have side effects associated with adjuvants, and they also face challenges such as the need for booster doses and stability issues. Biocompatible and biodegradable NPs could convey antigens to immune cells and overcome anthrax vaccination problems. Hence, the transfection of PLGA-encapsulated stable immunogenic domain 4 (PAD4) of *B. anthracis*’ protective antigen was carried out. Mice received a single dose of PAD4-NP or recombinant PAD4 control. In contrast to PAD4-NP, which produced a strong IgG response with a mix of IgG1 and IgG2a subtypes, PAD4-immunized mice only had a mild IgG response with a predominance of IgG1. While PAD4-NP provoked a mixed Th1/Th2 response, the PAD4 alone elicited a Th2 response. After a lethal *B. anthracis* spore challenge, the PAD4-NP-immunized animals outlived PAD4-immunized mice by 6 days. This research may lead to a safer and more efficient anthrax vaccine. This PAD4-NP was a single-dose anthrax vaccine that used a protective antigen and did not contain any adjuvants [174]. To prepare a nanovaccine aimed at preventing Brucella infection, *Brucella melitensis* LPS was used as an antigen. The vaccine stimulated both the humoral and cellular immune responses in immunized BALB/c mice. A novel mucosal adjuvant of a nontoxic water-in-oil nanoemulsion (NE) was developed. This formulation ruptures mucosal surfaces and allows for the loading of antigens into DCs, resulting in a proinflammatory response. The protective antigen from anthrax (rPA), conjugated with NE, was administered intramuscularly to guinea pigs and mice. This rPA-NE immunization effectively induced the production of serum anti-PA IgG antibodies, signifying a Th1-polarized immune response, as well as bronchial anti-PS IgA and IgG antibodies. When challenged intranasally with 10 and 100 times the LD_50_ dose of *B. anthracis* Ames strain spores (1.2 × 10^6^ and 1.2 × 10^7^ spores), guinea pigs that received the rPA-NE vaccine showed a survival rate of 70% and 40%, respectively. This study thus demonstrated the efficacy of intranasal administration of anthrax vaccination using the rPA-NE formulation [175]. Immune reactive TT-entrapped PLA and PLGA particles were synthesized for vaccination to prevent tetanus. Anti-TT antibody titers were raised by physically combining various-sized particles to immunize Wistar rats. Rats exhibited early, high antibody titers due to the physical mixing of nano- and microparticles. For over 5 months, anti-TT antibody titers from polymer particles encasing stabilized TT were higher than those from saline TT. Compared to double doses of alum-adsorbed TT, single-point vaccination with particles or physical mixes resulted in lesser antibody responses. Higher concentrations of anti-TT antibodies were detected 15 days postadministration of the NPs-alum vaccine. A single-point immunization with PLA microparticles and alum within 6 months elicited antibody titers over 50 μg/ml, unlike 2 administrations of alum-adsorbed TT. The entrapped antigen, proper vaccination technique, and adjuvant can generate long-lasting immune responses using polymer particles from single-dose vaccine formulations [176]. *Chlamydia trachomatis* is a sexually transmitted bacteria globally that causes a severe infection of sexual organs. For the development of the vaccine, the recombinant major outer membrane protein (rMOMP) was encapsulated in PLGA NPs. When PLGA-rMOMP was used to stimulate mouse primary DCs, it enhanced endosome processing, induced the production of Th1 cytokines (IL-6 and IL-12p40), and up-regulated the expression of MHC-II and costimulatory molecules (CD40, CD80, and CD86). Following PLGA-rMOMP immunization of BALB/c mice, they exhibited the production of functional antigen-specific serum IgG antibodies, along with enhanced development of CD4^+^ T-cell-derived effector (CD44^high^ CD62L^low^) and memory (CD44^high^ CD62L^high^) properties (Fig. 3A) [177].

BALB/c mice treated with either NPs alone or liposomes exhibited substantially reduced levels of T-cell activation compared to lymph node cells that were immunized with Mtb cell-wall-associated lipid-coated CS-NPs. Mtb cell-wall-associated lipid-bound CS-NP-stimulated cells in the lymph nodes and spleen release higher levels of Th1 and Th2 cytokines, as well as increased amounts of IgM, IgG, IgG1, and IgG2 compared to the control animals. Utilizing CS-NPs to effectively deliver Mtb lipids and enhance both cell-mediated and antibody responses represents a promising new approach [178]. *Streptococcus pneumoniae*, *Pseudomonas aeruginosa*, and *Aerobacter levanicum* polysaccharide antigens were intranasally administered in liposomes. sIgA titers against particular bacterial antigens may boost resistance to lung infections, including pneumonia. In each case, the intranasal vaccination with liposome-encapsulated antigen increased slgA specific for bacterial polysaccharides in the lungs. The intranasal polysaccharide antigen-containing liposomes produced a comparable lung slgA response with 1/30 the antigen needed for oral vaccination. Even with adjuvant, the oral vaccination requires substantial polysaccharide antigen dosages. *P. aeruginosa-*related pneumonia mortality was considerably reduced by intranasal vaccination. Therefore, liposome-based mucosal immunization targeting bacterial polysaccharide antigens increased lung slgA titers and reduced pneumonia susceptibility [179]. A bacterial outer membrane vesicle (OMV)-loaded dendritic mesoporous organosilica NPs (DMON)-based nanovaccine (LPS@DMON@OMV) was developed, where LPS was used as an adjuvant, against bacterial pneumonia, spread by *P. aeruginosa*. The LPS@DMON@OMV nanovaccine induced 180 times greater antibody titers than free OMV and increased cytotoxic T cells’ production to destroy pneumonia disease-causing bacteria in the body (Fig. 3B) [180]. Scientists developed the nanovaccine by combining PLGA NPs with LPS and OPS antigens, which activate the immune system to target *P. aeruginosa* at both cellular and humoral levels. Nanovaccine was synthesized via carbodiimide utilizing OPS–PLGA and LPS–PLGA conjugates. The conjugated nanovaccine, pure OPS, and LPS antigens were delivered to BALB/c mice thrice, with a 2-week interval between each administration. The enzyme-linked immunosorbent assay (ELISA) test indicated that antibodies against OPS–PLGA or LPS–PLGA conjugates were higher than those against pure antigens, encompassing IgG, IgG1, IgG2b, IgG2a, IgG3, IgM, and IgA. The opsonophagocytosis test results indicated that the anti-LPS–PLGA antibodies produced had a superior and more pronounced effect relative to the other groups. The LPS–PLGA compound also prevented *P. aeruginosa* infection in mice better than the other groups [181].

Streptococcal disease occurs by infection of *Streptococcus iniae* and *Streptococcus agalactiae*, mainly in aquaculture/farming. Based on the prediction of multiple sequence alignment and B-cell antigenic epitope studies of the Sip and Srr sequences, a multi-epitope subunit vaccine known as Sip-Srr (SS) was created. Moreover, the rSS protein was coupled with modified bacterial nanocellulose (BNC) to create the BNC-rSS nanocarrier vaccination system against *Streptococcus* sp. infection. BNC-rSS nanovaccine-immunized tilapia fish showed a higher survival rate than rSS-immunized tilapia fish against streptococcal disease [182]. To elicit lasting humoral and cellular responses, liposomes incorporating the *Streptococcus pyogenes* peptide antigen J8, diphtheria toxoid, and the immunostimulatory glycolipid 3D(6-acyl) PHAD were intranasally delivered. The study shows the essential functions of cellular responses, including CD4^+^ T cells, IL-17, neutrophils, and macrophages, in vaccine-induced mucosal immunity in IL-17 knock-out BALB/c mice [183]. An oral liposome vaccination against *Helicobacter pylori* infection was developed with this fusion peptide and tested in BALB/c mice. The B subunit of cholera toxin (CtUBE) and the B subunit epitope of *H. pylori* urease were combined into a peptide and expressed in *E. coli*. BALB/c mice given the CtUBE liposome vaccine intragastrically displayed marked disease resistance in preventive and therapeutic immunization protocols, increasing specific anti-urease mucosal IgA and blood IgG levels. Owing to its prolonged immunogenicity, the fusion peptide CtUBE may serve as an antigen in a prospective oral vaccination for *H. pylori* infection [184]. A TLR-independent immunomodulator, such as a synthetic mycobacterial glycolipid, was delivered using cationic liposomes, which stimulated robust and protective Th1 and Th17 responses in adult mice against Ag85B-ESAT-6, an essential mycobacterial fusion protein. CAF01 satisfactorily achieved the preclinical requirements as a novel TB vaccine to be utilized in infancy and childhood due to its powerful adult and neonatal adjuvanticity coupled with exquisite yet delayed DC’s uptake and activation, in vivo. Adjuvants like CAF01 showed great promise as a component of vaccinations for childhood and adolescent diseases [185]. Human listeriosis is caused by the foodborne intracellular bacterium *Listeria monocytogenes*. A novel peptide antigen has been identified from in vitro cells infected with *L. monocytogenes*. The cells displaying the peptide antigen were presented by the MHC-I receptor and have been utilized as vaccination antigens. Cells infected with *L. monocytogenes*, EGD, possessed 42 unique antigens, among which 5 were recognized as oligopeptide ABC transporters or periplasmic oligopeptide-binding proteins (OppA): LMON_0134, LMON_0149, LMON_2115, LMON_2272, and LMON_2584 in EGD. MHC Class I peptides represented 3 of these: LMON_0134, LMON_0149, and LMON_2272. Each peptide’s mRNA was synthesized in vitro. Single and combined mRNA-loaded cationic LNPs containing the immunopotentiator α-galactosylceramide were used for vaccination formulation. The combination vaccine formulation of LLO_E262K and LMON_2272 demonstrated a marked reduction of bacterial titer in the liver and spleen, and it also activated CD8^+^ T cells in vivo through the secretion of IFN-γ cytokines by APCs. [186].

Mucosal vaccination against *Burkholderia cepacia* complex (Bcc), an opportunistic microbe, employed an NE adjuvant and a new *B. cenocepacia-*based OMP antigen. CD-1 mice received *B. cenocepacia* OMP antigen intranasally combined with NE or phosphate-buffered saline (PBS). Vaccinated mice had strong serum IgG, and mucosal secretory IgA immunological response antibodies neutralized *B. cenocepacia* and *B. multivorans*. Mice immunized could avoid *B. cenocepacia* infection in their lungs [187]. Pneumococcal capsular polysaccharides (CPS14) polymerized on the surface of *E. coli*-derived membrane vesicles used as nanovaccine protect BALB/c mice from pneumococci and produce higher antigen-specific IgG and IgM response. The lung and blood humoral immune responses elicited by CPS14^+^MV persisted for 1 year. Mice of different ages showed marked efficacy with the CPS14^+^MV vaccine. Immunization elicited robust CPS14-specific IgG that bound to the pneumococcal cell surface, including in aged mice [188].

### NP-based vaccines for parasitic infections

**Fig. 4.**
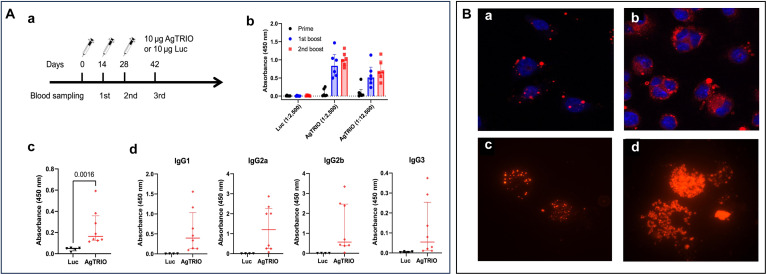
(A) AgTRIO mRNA-LNP immunization generates a robust IgG response against recombinant AgTRIO and salivary gland extracts. (a) Experiment scheme showing groups of C57BL/6 female mice injected with 10 μg of AgTRIO mRNA-LNP or control mRNA (Luc mRNA-LNP), and boosted twice, at 2-week intervals. (b) Two weeks after each immunization, mice were bled. 1:2,500 and 1:12,500 dilutions of sera were examined for AgTRIO-specific IgG antibodies by ELISA using recombinant AgTRIO as the antigen. (c) 1:2,500 dilution of sera collected after the final was examined for AgTRIO-specific IgG antibodies against salivary gland extracts by ELISA (median ± IQR, *P* < 0.05 using the Mann–Whitney *U* test). (d) 1:2,500 dilution of sera was used to determine AgTRIO-specific IgG1, IgG2a, and IgG2b. 1:500 dilution of sera was examined for AgTRIO-specific IgG3 antibodies. This figure is reproduced under the terms of the Creative Commons CC BY license. Copyright (2023) Nature [189]. (B) NPs uptake by antigen-presenting cells. (a and b) Confocal microscopy images of J774 macrophages after incubation for 2 h with NR-MPLA-NPs (a) and CS-NR-MPLA-NPs (b). The samples were counterstained with the Hoechst 33342 fluorescent dye (blue). (c and d) Fluorescence microscopy images of DCs after incubation for 24 h with NR-MPLA-NPs (c) and CS-NR-MPLA-NPs (d). Reprinted with permission from Leroux et al*.* [192]. Copyright (2023) Elsevier.

Mosquitoes carrying *Plasmodium* inject saliva into vertebrate hosts to induce malaria. C57BL/6 mice safeguarded against *Plasmodium* infection via active or passive immunization utilizing the AgTRIO saliva protein derived from mosquitoes. Following immunization with AgTRIO mRNA-LNP, mice had a pronounced immunological response characterized by the production of protective AgTRIO IgG2a isotype antibodies. Mice vaccinated with AgTRIO mRNA-LNP exhibited substantially lower levels of *Plasmodium* hepatic infection and improved survival rates when exposed to mosquitoes infected with *Plasmodium berghei*, relative to the control group. After 6 months, the immune response to AgTRIO decreased, whereas more mosquito bites increased IgG1 and IgG2a isotypes (Fig. 4A) [189]. Nucleoside-modified Pvs25 mRNAs loaded into LNP were also used as a nanovaccine, and an in vivo study on mice produced a more robust and enduring functional immune response. After 7 months, followed by the second dose, the antibodies produced by the vaccine were still effective in preventing the transmission of *P. vivax* in direct membrane feeding assays [190]. Rabbits were immunized with cholera toxin (CT) along with a synthetic 16-residue peptide conjugated to albumin, derived from the circumsporozoite protein of *Plasmodium falciparum*. CT was immunogenic but fatal, whereas the combination of the malaria peptide and albumin was nonimmunogenic. CT lost its toxicity and gained antigenicity when attached to liposomes containing ganglioside GMI. Thus, liposomes could reduce the toxicity of hazardous antigens. High titers of malaria peptide-specific antibodies were produced by liposomal combinations of the peptide and albumin, but not albumin antibodies. These antibodies recognized natural circumsporozoite protein (CS protein). The 3 liposome adjuvants, lipid A, and 2 different types of lipophilic muramyl di-peptide performed well. Because of the synthetic *P. falciparum* CS peptide’s ability to switch between being nonimmunogenic and immunogenic and the fact that liposomes work as “toxoids” for CT, it is possible that liposomes can be helpful as both antigen carriers and adjuvants for vaccinations when dealing with dangerous or poorly immunogenic substances [191].

*E. granulosus*, a cestode parasite responsible for cystic echinococcosis in humans and several animals, predominantly resides in canines. A PNP-based oral vaccine for *E. granulosus* was developed and administered to dogs as enteric-coated capsules. Two recombinant protein antigens EgTrp (*E. granulosus tropomyosin*) and EgA31 (*E. granulosus paramyosin*) encapsulated into biodegradable PNPs, along with the Monophosphoryl lipid A addition, as an adjuvant, improved the delivery and stimulated a shielding mucosal immune response. CS was applied to the NP’s surface to enhance its adhesion to the gut mucosa and its ability to transport antigens. In vitro experiments showed that CS-coated NPs enhanced internalization and penetration in murine macrophages and DCs as well as Caco-2 cells (Fig. 4B) [192].

## Safety of the NPs for Vaccine Formulation

The primary issue with NPs is their potential cytotoxicity, which can adversely affect cells. To address this problem, researchers assess the toxic concentrations of NPs on various cell lines, typically over 24 or 48 h of incubation. For example, the modified Au nanorods, PDDAC-Au, and PEI-Au NR do not have severe toxicity in HEK-293 cell lines after 24- and 48-h treatment [142]. In a study, immature (day 5) DCs were exposed to varying concentrations of the single-wall carbon nanotube 4-formyl-benzoate (SWNT–4FB) scaffold, with concentrations reaching up to 100 μg/ml. The aim was to evaluate any potential harmful effects of the SWNTs. An ATP lite assay for cellular ATP generation, an Alamar blue assay for cellular metabolic activity, and a bisbenzimide stain for cellular apoptosis were employed to assess cellular viability. In all 3 tests, administration of SWNT–4FB at concentrations up to 100 μg/ml exhibited no dose-dependent cytotoxicity on immature DCs [56]. HepG2 cells were exposed to CS NPs, averaging 167.6 nm in diameter, over a 48-h period at concentrations ranging from 5 to 100 μg/ml. A 12% reduction in cell viability at a concentration of 100 μg/ml, relative to an IC_50_ value of 239 μg/ml, demonstrates that HepG2 cells have considerable resistance to CS NPs. Chicken embryo kidney (CEK) cells were cultured in Dulbecco’s Minimum Essential Media (DMEM), then diluted, and subsequently placed into 96-well plates (1 × 10^5^ cells/well). NDV-CS-NPs (100 μl; 1.5 μg/ml stock) were introduced, followed by incubation of the plates at 37 °C for a duration of 48 h. The survival rate of CEK cells was 91.5% ± 5.3%, and there were no significant changes in cell morphology compared to the control cells, suggesting a low level of cytotoxicity associated with NDV-CS-NPs [141]. CS NP-based cell viability assay was done on a mouse macrophage cell line, and 50% of cells died in a 75 μg/ml concentration of CNP [178].

Cytotoxicity of PLA-NPs had been assessed in A549 cells to varying doses of PLA-NPs (2, 20, 100, and 200 μg/ml) for distinct durations (6, 24, 48, and 72 h). The viability was unaffected, as indicated by the conversion of 3-(4,5-dimethylthiazol-2-yl)-2,5-diphenyltetrazolium bromide (MTT) into formazan throughout all exposure settings [69]. Another study assessed the cytotoxicity of loaded γ-PGA and unloaded γ-PGA NPs in vitro using HL-60 cells. After 24 to 72 h of incubation, the proliferation and viability of HL-60 cells were examined at the concentrations of γ-PGA and empty NPs. Even at high doses (1 mg/ml), cellular viability was maintained at values greater than 90% for both γ-PGA and empty NPs [68]. Researchers used mouse splenocytes to investigate the cytotoxicity of nanogels, as these splenocytes encompass a diverse range of immune cells, including B and T lymphocytes, monocytes/macrophages, and NK/NKT cells, which are crucial for immune responses induced by vaccines. Nanogels at concentrations between 10 and 60 mg/ml had no marked effect on increasing cell mortality after 24 h of incubation. At 80 to 100 mg/ml doses, AP-CC and AP-SS nanogels exhibited modest cytotoxicity, resulting in the death of only 15% to 25% of cells [86]. The MTT assay was utilized to assess the cytotoxicity of PLGA-PEG NPs. The MTT results were obtained from cells exposed to spherical and needle-shaped NPs at doses ranging from 5 to 250 μg/ml over a 24-h period. At elevated concentrations (250 μg/ml), the cytotoxicity of needle-shaped NPs increased with the concentration of NPs; at lower doses (approximately 93% cell viability at 5 μg/ml), it decreased to roughly 56%. Conversely, spherical-shaped NPs demonstrated no cytotoxicity, with cell survival approximately 84% at this dose. A similar discovery was achieved using HeLa cells [193]. Another study on the NSC-3 cell line showed no cytotoxicity of PLGA NPs in 0.4 mg/ml [73].

To determine the cytotoxicity of LNPs, cell seeding was performed in a 96-well microplate with 25,000 HL60, 10,000 NB4, 7,000 A549, and 5,000 NIH3T3 cells. Each well received 50 μl of cell suspension, with each condition reproduced across 6 wells. Background measurements were performed in 6 wells using 100 μl of the appropriate medium. To evaluate the toxicity of 3D cultures, 1,000 A549 and HL60 cells were inoculated per well onto a U-bottom 96-well plate coated with 1.5% agarose. After 2 to 3 days, cationic LNP-siRNA (0 to 128 μg/ml) was introduced to the spheres and incubated for 48 h at 37 °C with 5% CO_2_. All 3 LNP-siRNA formulations showed no discernible deleterious effects on HL60 and NB4 cells at concentrations up to 128 μg/ml, which is far exceeding the typical dosage for gene expression. Cell viability in HL60 cells subjected to 512 μg/ml ALNP, NLNP, and CLNP was 68%, 74%, and 92%, respectively. The NB4 suspension cell line exhibited 91%, 61%, and 85% viability following treatment with ALNP, NLNP, and CLNP, respectively. The administration of CLNP at a concentration of 128 μg/ml resulted in significant toxicity in adherent cell lines (NIH3T3 and A549), with viability assessed at 33% and 54%, respectively. This CLNP content substantially exceeds the norm. In A549 cells, 512 μg/ml of ALNP and NLNP did not diminish cell viability (71% and 77%, respectively) [194].

The CCK-8 assay was employed to assess the cytotoxicity of EVs in bone marrow-derived dendritic cells (BMDCs). Femurs and tibias from mice were used to collect BMDCs, which were then cultivated in RPMI 1640 complete medium supplemented with GM-CSF (20 ng/ml) and IL-4 (10 ng/ml). To evaluate the cytotoxic effects of EVs on BMDCs, 96-well plates were seeded with 1 × 10^5^ cells per well, incubated for 12 to 16 h, and then treated with EVs, mesoporous silica nanoparticles (MSNs), and EV/ICG/MSN complexes at escalating concentrations for 24 h. No reduction in cell viability was seen with any of the 3 EV types, as anticipated [195].

In addition to evaluating in vitro cytotoxicity, it is essential to examine in vivo biodistribution, systemic tolerance, and the clinical implications of nanovaccine safety. The distribution and elimination of NPs within tissues are primarily determined by their physicochemical properties, such as size, composition, and surface chemistry. Preclinical investigations indicate that NPs, with dimensions ranging from 20 to 100 nm, are capable of migrating to lymph nodes after being administered via intramuscular injection. This minimizes systemic exposure and enhances immune targeting [26,27]. The distinct safety profile of biodegradable PNPs such as PLGA is noteworthy. The endogenous metabolism of lactic and glycolic acid resulting from PLGA hydrolysis enhances systemic biocompatibility [87]. The localized acidity that occurs during breakdown can influence inflammatory responses, which may vary based on the antigen load and formulation. Cationic polymers such as PEI are beneficial for nucleic acid complexation and intracellular transport; however, they can adversely affect membranes and mitochondria in a dose-dependent manner [196]. The data indicate that the selection of polymeric systems requires a careful balance between biocompatibility and delivery efficacy.

Preclinical animal studies indicate that inorganic NPs, such as AuNPs utilized for multivalent antigen presentation, exhibit short-term tolerance. However, their nonbiodegradable nature raises concerns regarding long-term persistence and potential bioaccumulation [197].

Multiple trials indicate that there is no acute toxicity at vaccine-relevant dosages; however, long-term safety data are comparatively limited when compared with lipid-based platforms. The uptake of mononuclear phagocytes can result in the accumulation of particles in the liver and spleen upon entering the systemic circulation. Studies on the biodistribution of LNPs reveal that pathways dependent on apolipoprotein E facilitate a preferential accumulation in the liver [198]. The results highlight the importance of a systematic strategy in material design and management to minimize off-target organ deposition. The ionizable LNPs represent the most thoroughly clinically assessed nanovaccine technology to date. Unlike permanently cationic lipids, ionizable lipids undergo protonation in endosomes while remaining neutral at physiological pH. This characteristic reduces systemic toxicity and improves effective cytosolic transport [199]. Studies on structure–activity optimization reveal that lipid composition directly affects antigen expression and inflammatory responses. Improved formulations in vivo lead to enhanced immunity and temporary innate activation [28].

Phase III clinical trials utilizing mRNA vaccines encapsulated in ionizable LNPs provide the strongest evidence regarding the safety of nanovaccines. Further studies revealed no long-term organ damage, and extensive trials involving BNT162b2 and mRNA-1273 showed only transient local and systemic reactions [200,201]. Multiple hypersensitivity events that may be linked to polyethylene glycol components were confirmed through post-authorization monitoring; however, the overall benefit–risk profile continued to be favorable [202].

## Conclusion and Future Perspective

Nanotechnology has substantially widened the domains of research, particularly in vaccines. The small size and large surface area of vaccine nanoformulations provide an edge over the traditional vaccination platforms. Therefore, NPs are potential candidates for vaccine delivery. The NP-based vaccine formulation has proven effective against numerous diseases. Many new and re-emerging illnesses possess the capacity to multiply, shield antigens, and provoke robust immune responses, rendering them more effective than conventional immunizations. Nanovaccines provide a versatile, efficient, and rapidly deployable approach to managing infectious diseases, ranging from polymer- and liposome-based vaccines for TB and malaria to mRNA–LNP vaccines for viral pandemics. Interdisciplinary collaboration and innovative regulatory approaches will be crucial for achieving their complete translational potential. The development and use of a wide variety of NPs as delivery systems or immune potentiators have improved antigen stability, enhanced antigen processing, and immunogenicity. Nanovaccines against viral infections provide robust protection against viruses such as SARS-CoV-2, HIV, influenza, and hepatitis by boosting both humoral and cellular immune responses. LNPs were successfully utilized in the COVID-19 mRNA vaccine, demonstrating their potential for rapid vaccine development. Engineering NPs and other designed NPs improve immune responses against drug-resistant bacterial infections such as *H. pylori*, pneumococcus, and TB. They strengthen mucosal immunity and offer lifetime protection.

Interestingly, the NPs excel as dual agents: they scaffold and stabilize fragile antigens, amplify and process them, and provide immunogenicity via codelivery of cytokines (e.g., GM-CSF) and TLR agonists, thereby turbocharging APC activation. Furthermore, the use of nanovaccines that harness the mucosal frontiers yields to oral/intranasal formats, while transdermal and inhalable nanovaccines break injection barriers. This is advantageous for a widespread patient population and also provides equity in resource-scarce settings. However, the clinical translation of nanovaccine formulations requires careful evaluation of NP biocompatibility, high-capacity adjuvant loading to influence adaptive immunity, controlled payload release, and postdelivery behavior. Therefore, for a better future of nanovaccines, interdisciplinary works spanning materials science, immunology, and adaptive regulation will be required at their zenith to develop nanovaccines as the foundation for next-generation immunization paradigms.

In recent times, new horizons such as artificial intelligence-assisted platforms will hyper-personalize formulations. These in silico platforms will predict epitope drift via real-time genomic surveillance and auto-adapting LNP payloads for universal viral protection. This will help prevent future pandemics. Other future endeavors for nanovaccines could include saRNA encapsulated in VLPs alongside mucosal adjuvants such as flagellin to facilitate noninvasive, inhalable vaccination initiatives for airborne pathogens like TB and influenza, while thermostable PLGA matrices for nanovaccine manufacturing can enhance distribution in low- and middle-income countries. Moreover, biohybrid NPs in conjunction with engineered bacteriophages can modulate immune responses against antimicrobial-resistant superbugs. Additionally, faster regulatory approvals and blockchain-verified manufacturing data will connect global factories through open IP sharing. This makes nanovaccines affordable for everyone, transforming them into vital public health tools against deadly outbreaks. Ultimately, nanovaccine development aims to not only eradicate pathogens but also foster a united human front against future microbial threats and refine global immunization. We hope that the NP-based vaccine will be a promising candidate for the development of next-generation vaccines.
