# Supplementary material for: Nanoparticle-Based Antigen Delivery: Advancing Immunization Strategies against Infectious Pathogens
Source: Biomater Res. 2026 Jul 3;30:0385. doi: 10.34133/bmr.0385 (PMC13329038; doi:10.34133/bmr.0385)
Supplement: Supplementary 1 — Fig. S1 [file bmr.0385.f1.docx]

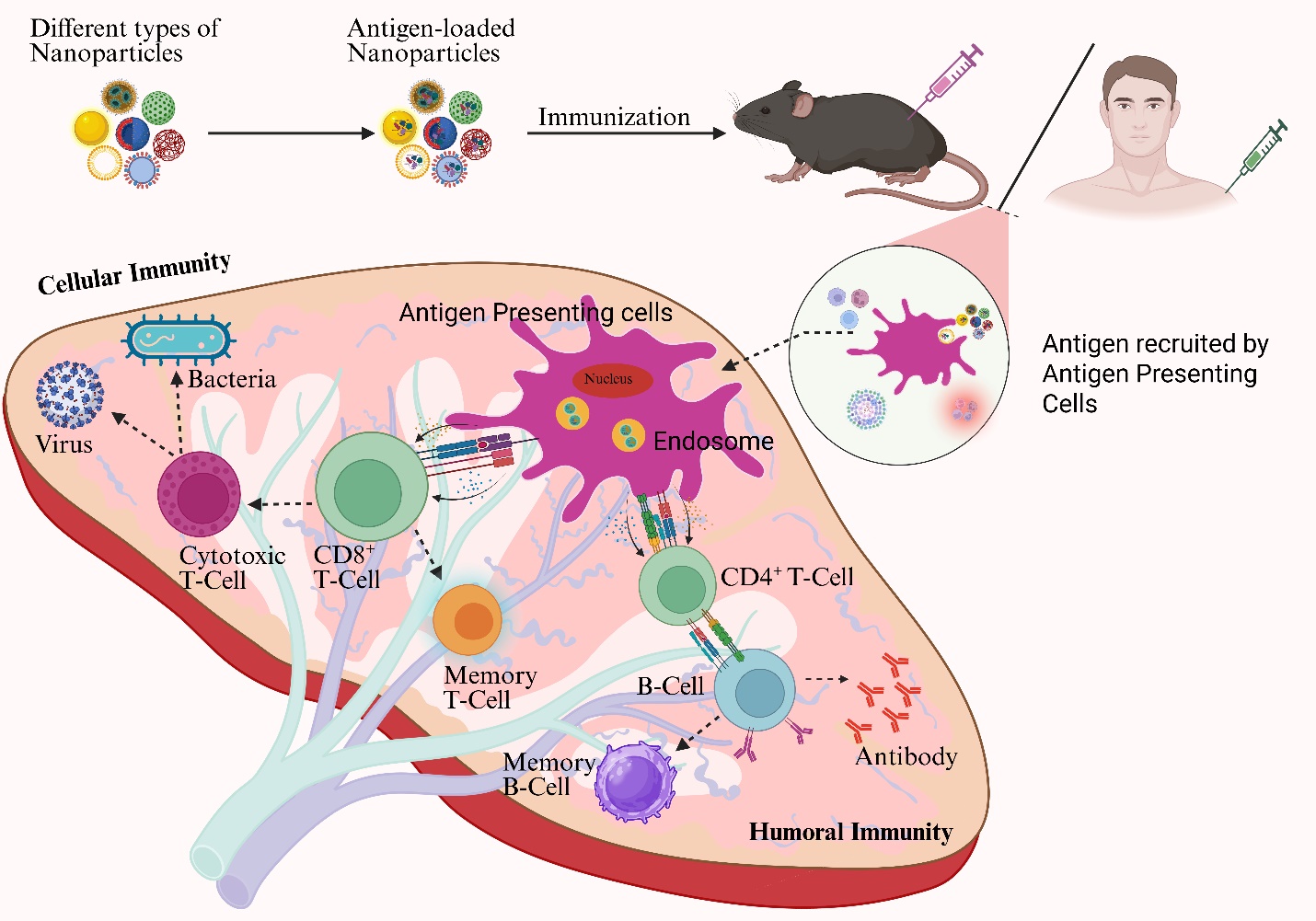


Figure S1: Schematic representation of the administration of antigen-carrying nanoparticles, internalization in immune cells, and activation of cellular and humoral immunity.
